# Closed-Loop Multi-Objective Optimization for Receptor-Selective Cell-Penetrating Peptide Design

**DOI:** 10.1021/acs.jcim.6c01267

**Published:** 2026-09-10

**Authors:** Iori Yamahata, Hidetomo Yokoo, Yosuke Demizu, Teppei Shimamura, Shuto Hayashi

**Affiliations:** † Laboratory for Data-driven Biology, Nagoya University Graduate School of Medicine, 65 Tsurumai-cho, Showa-ku, Nagoya, Aichi 466-8550, Japan; ‡ Department of Computational and Systems Biology, Division of Biological Data Science, Medical Research Laboratory, Institute of Integrated Research, 13290 Institute of Science Tokyo, 1-5-45 Yushima, Bunkyo-ku, Tokyo 113-8510, Japan; § Division of Organic Chemistry, National Institute of Health Sciences, 3-25-26 Tonomachi, Kawasaki-ku, Kawasaki, Kanagawa 210-9501, Japan; ∥ Graduate School of Medical Life Science, Yokohama City University, 1-7-29 Suehiro-cho, Tsurumi-ku, Yokohama, Kanagawa 230-0045, Japan; ⊥ Graduate School of Medicine, Dentistry and Pharmaceutical Sciences, Okayama University, 1-1-1 Tsushima-naka, Kita-ku, Okayama 700-8530, Japan; # Innovation Center of NanoMedicine, Kawasaki Institute of Industrial Promotion, 3-25-14 Tonomachi, Kawasaki-ku, Kawasaki, Kanagawa 210-0821, Japan

## Abstract

Cell-penetrating peptides (CPPs) can deliver diverse cargoes into cells. However, designing CPPs with receptor-selective interaction profiles remains difficult because interactions with individual cell-surface components cannot be tuned independently. Here, we developed a closed-loop in silico framework for receptor-selective CPP design, in which receptor interactions are formulated as explicit objectives in a multi-objective optimization problem. We first constructed a CPP-like candidate library by using a sequence generative model fine-tuned on known CPPs. The framework then evaluated candidate peptides by receptor-wise docking, molecular dynamics simulations, and MM/GBSA to compute receptor-wise binding scores as computational proxies for receptor-associated interactions. These scores were used iteratively to propose subsequent candidates by multi-objective Bayesian optimization. Applied to a CXCR4/NRP1 design setting, the framework identified candidates with more favorable predicted interaction profiles characterized by higher CXCR4 binding scores and lower NRP1 binding scores. We selected 10 peptides from the computationally identified candidates for cell-based imaging and found that 4 showed higher enrichment in CXCR4-positive regions than in NRP1-positive regions under the tested conditions. These results show that the proposed framework provides a practical in silico approach for designing CPPs with receptor-selective interaction profiles.

## Graphical abstract

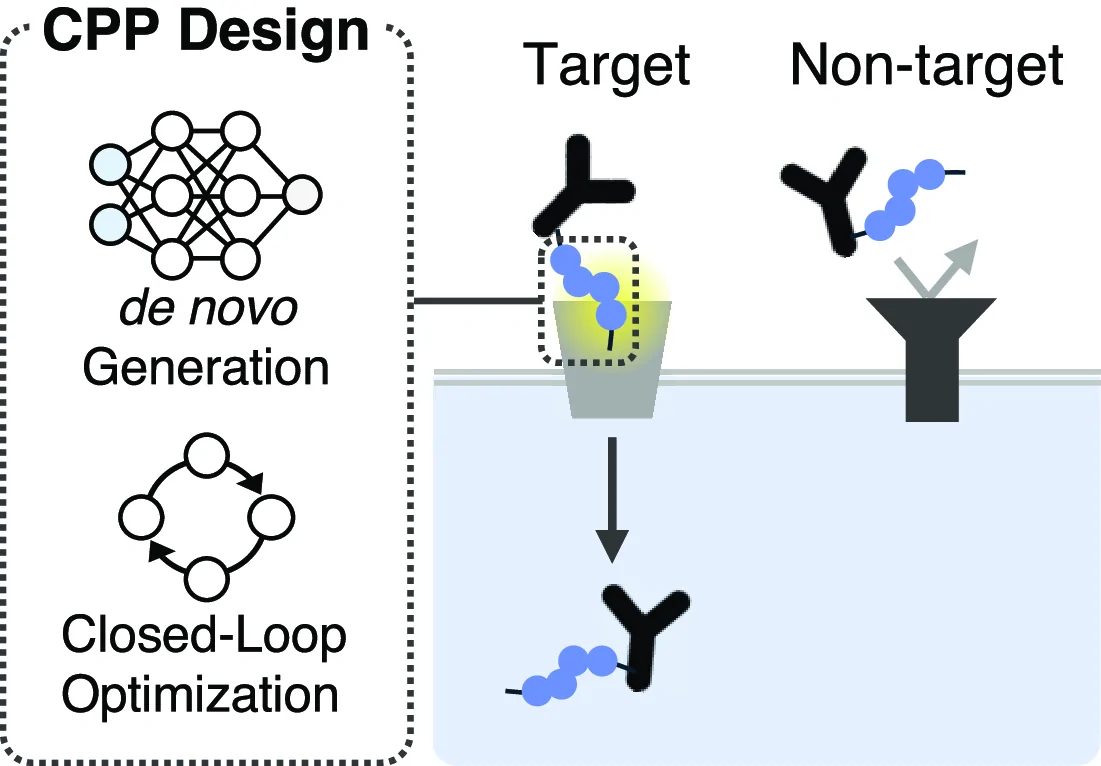

## Introduction

Cell-penetrating peptides (CPPs) are promising molecules for intracellular delivery because they can transport diverse cargos, including proteins, nucleic acids, and small molecules, into cells.^26,36^ Their uptake involves multiple pathways and is often shaped by concurrent interactions with cell-surface receptors and glycans.^5,33^ Since interactions with individual cell-surface components cannot be tuned independently, it is difficult to achieve receptor-selective uptake and avoid off-target uptake.^14,19,33^

Recent advances in CPP research have promoted both computational prediction and mechanistic evaluation of cellular uptake. On the prediction side, expanded CPP databases, deep-learning-based screening methods, and prediction models have enabled large-scale evaluation of CPP-like sequences.^3,17,34^ On the simulation side, molecular dynamics has been used to evaluate AI-generated CPPs and to analyze CPP–membrane interactions and the behavior of drug-conjugated CPPs.^18,41^ In contrast, selective CPP design has often relied on introducing or modifying sequence motifs to enhance interaction with a desired target molecule.^11,25,29,30,35^ However, these approaches typically optimize CPP-likeness, uptake-related properties, or target interaction strength in isolation. Explicit treatment of target and counter-objective receptor interactions as separate design objectives remains limited. As a result, computational frameworks that evaluate receptor-wise interaction profiles and use them to guide sequence optimization under competing cell–surface interactions are still scarce.

Here, we present a closed-loop in silico framework for receptor-selective CPP discovery (Figure 1), in which receptor interactions are formulated as explicit objectives in a multi-objective optimization problem. The resulting framework links CPP-like sequence generation, receptor-specific evaluation, and Bayesian optimization within a closed-loop design cycle, enabling candidate exploration based on receptor interaction trade-offs rather than on a single-receptor objective.

**Figure 1:**
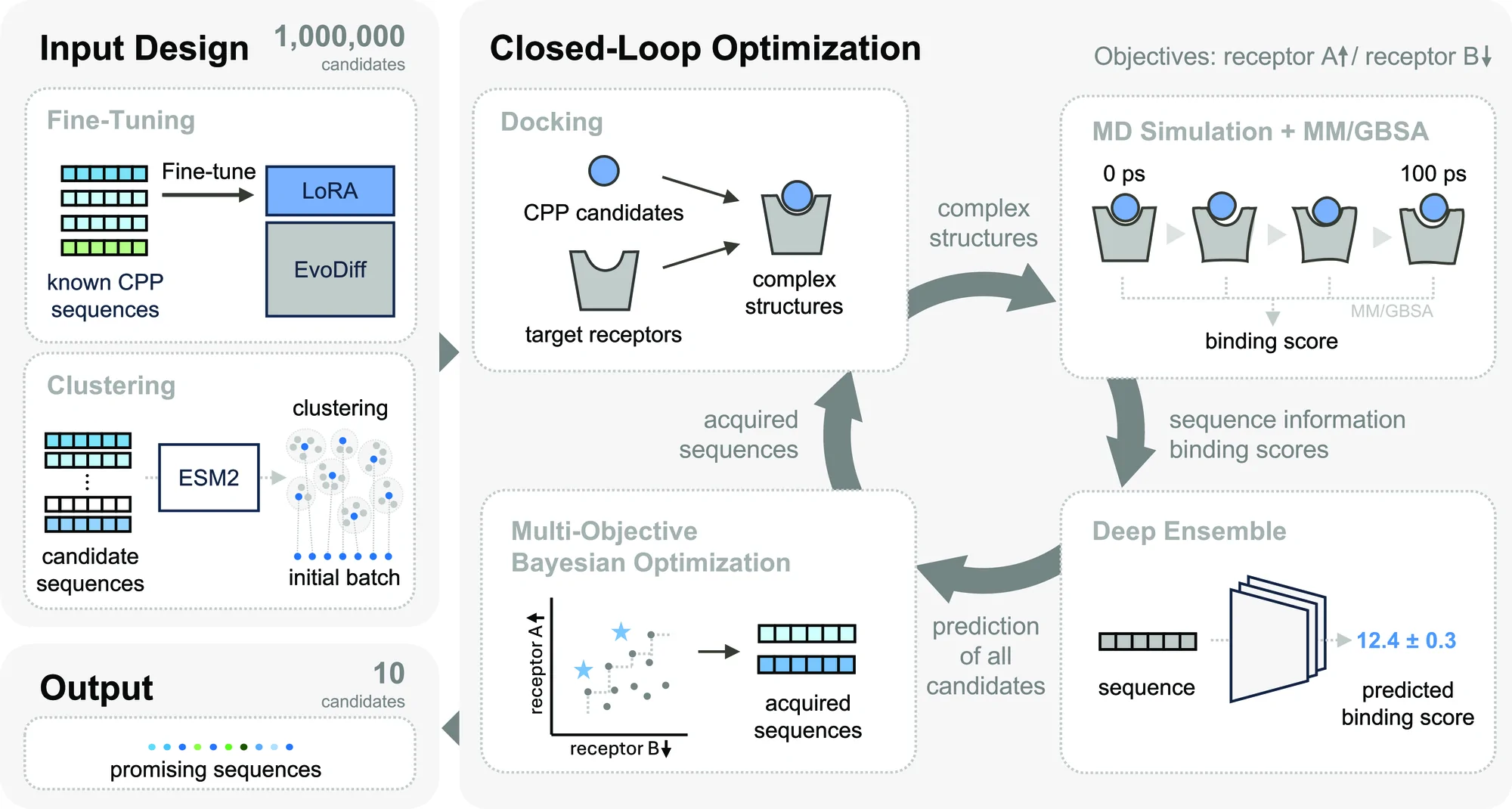
Schematic diagram of the closed-loop multi-objective optimization framework for receptor-selective CPP design. A total of 1,000,000 candidate sequences are generated using a sequence generative model (EvoDiff + low-rank adaptation (LoRA)) fine-tuned on known CPP sequences, and an initial candidate set is constructed by clustering feature vectors from a protein language model. In the optimization loop, receptor-wise binding scores are evaluated for selected peptide–receptor complexes using docking, MD simulations, and MM/GBSA. The deep ensemble surrogate model is then updated with the accumulated data, and multi-objective Bayesian optimization is used to propose sequences from the 1,000,000-sequence candidate library for evaluation in the next cycle. Through iterative repetition of this cycle, candidate peptides with receptor-selective predicted interaction profiles are identified.

The methodological contributions of this work can be summarized in three ways. First, we formulate receptor-selective CPP design as a multi-objective optimization problem. By simultaneously treating the maximization of the target-receptor score and the minimization of the counter-objective-receptor score as independent objectives, we explicitly explore relative selectivity between receptors through candidates on the Pareto front. Such selectivity cannot be directly targeted in a single-objective formulation. Second, we constructed a unified receptor-wise binding score. By combining docking pose selection based on receptor-side interaction regions with MM/GBSA binding free energies normalized by the number of residues, we define an evaluation metric that is robust to variation in sequence length and binding location and that allows fair comparison across structurally distinct receptors. Third, we developed a closed-loop optimization architecture that makes computationally expensive physics-based evaluation practical at scale. A CPP-like sequence generative model based on a fine-tuned protein sequence generative model,^2,16^ a deep ensemble surrogate model^22^ that predicts the mean and variance of the receptor-wise binding score, and a noise- and batch-aware multi-objective Bayesian optimization acquisition function (qNEHVI)^8^ are integrated into a single iterative cycle. Thus, the limited evaluation budget can be concentrated on the most promising candidates that are expected on the Pareto front.

We applied this framework to an CXCR4/NRP1 model system. In this setting, CXCR4 was treated as the target receptor and NRP1 as the counter-objective receptor. CXCR4 has been associated with leukemia-related extramedullary infiltration,^7^ whereas NRP1 is expressed in endothelial cells and various tumor cells and contributes to angiogenesis.^15,20^ This model setting represents a design scenario in which CXCR4-associated interaction is enhanced while NRP1-associated interaction is reduced.^33,39^ In this setting, the framework identified candidates with more favorable predicted CXCR4 and NRP1 interaction profiles. Confocal imaging of 10 computationally selected sequences in HeLa cells further revealed 4 of the 10 peptides with significantly higher enrichment in CXCR4-positive regions than in NRP1-positive regions. These results support closed-loop multi-objective in silico optimization as a useful framework for receptor-selective CPP design under competing cell–surface interaction constraints.

## Methods

### Overview

The overall framework is summarized in Figure 1 and consists of an input-design phase followed by a closed-loop multi-objective optimization phase. In the input-design phase, a CPP-like candidate library is constructed by sampling from a sequence generative model that has been fine-tuned on known CPP sequences, and an initial subset of this library that broadly covers the candidate space is selected to seed the optimization phase. The closed-loop optimization phase then proceeds as a repeating cycle of four operations. Each candidate sequence is docked against both the target and the counter-objective receptors, and a receptor-wise binding score is computed by a short molecular dynamics simulation, followed by MM/GBSA after pose filtering. The resulting sequence–score pairs are added to the training set of a deep ensemble surrogate model, which is retrained to predict the mean and variance of the binding score for each receptor. A multi-objective Bayesian optimization acquisition function then proposes the next batch of sequences from the candidate library for physics-based evaluation, and the proposed sequences enter the subsequent round of evaluation. The loop is terminated according to a stopping criterion based on the consistency of the top-ranked sequences, after which a final ranking is computed over all evaluated sequences and a small set of top candidates is selected for downstream experimental validation.

The architectural choices outlined above are guided by three design principles: first, exploration is restricted to a CPP-like region of sequence space because the combinatorial space of amino acid sequences is too large for random search or local motif grafting to cover the relevant region; we therefore use a generative model fine-tuned on known CPPs to encode CPP-likeness as an implicit prior over the candidate library. Second, the receptor-wise binding score used as an objective is constructed to be comparable across sequences of different lengths and across structurally distinct receptors. Docking poses are filtered against receptor-side interaction regions to ensure the biological plausibility of the binding location. In addition, the MM/GBSA-derived binding free energy is normalized by the number of residues to remove the length-dependent bias. Third, the cost of physics-based evaluation is allocated where it is most promising: a deep ensemble surrogate^22^ provides receptor-wise score predictions together with their uncertainties, and a noise- and batch-aware multi-objective acquisition function (qNEHVI)^8^ uses these predictions to select sequences that are expected to expand the Pareto front in subsequent cycles. These three principles operate jointly within a single closed loop rather than in isolation, and each plays a distinct role in making receptor-selective CPP designs tractable in practice. The remainder of this section describes each component of the framework in turn, beginning with the sequence generative model used to construct the CPP-like candidate library.

### CPP Sequence Generation Model and Candidate-Library Construction

#### Fine-Tuning of the Sequence Generative Model

To enable efficient exploration in the vast peptide sequence space, a CPP-like candidate library is constructed by fine-tuning a sequence generative model to known CPPs. EvoDiff OADM640M,^2^ an autoregressive protein sequence generative model, is employed as the base model. A total of 1082 CPP sequences from CellPPD^12,13^ are used as the training data set. Fine-tuning is performed using LoRA^16^ on the CPP data set. The fine-tuned model is then used to generate 100,000 sequences for each length from 9 to 18 residues, yielding a library of 1,000,000 CPP-like candidate sequences.

#### Selection of the Initial Batch for the Closed-Loop Optimization

An initial batch is selected from the library of the 1000 000 candidate sequences to reduce redundancy while broadly covering the CPP-like candidate space. Embedding vectors are computed for each sequence using ESM2 (esm2_t33_650M_UR50D),^24^ and *k*-means clustering is performed on residue-averaged embeddings. The number of clusters is set to 10,000, and the sequence with the smallest Euclidean distance to each cluster centroid is selected as its representative. The resulting 10,000 sequences are used as the initial set for the subsequent optimization loop.

#### Closed-Loop Multi-Objective Optimization

The objective of the closed-loop optimization is to identify CPP candidates with favorable predicted score profiles from the pregenerated library of the 1,000,000 candidate sequences. In each cycle, selected candidates are evaluated, the prediction model is updated with the newly obtained data, and new candidate sequences are proposed for evaluation in the next cycle. As a practical stopping criterion, the loop is terminated when the set of the top 50 sequences remains unchanged for 10 consecutive cycles.

#### Docking and Selection of Docking Poses

To generate peptide–receptor complex structures, DiffDock^6^ is used. Within each cycle, 20 docking poses are generated for each peptide–receptor pair. The poses are then filtered to remove physically or biologically unrealistic structures based on three criteria: intrapeptide clashes, peptide penetration into the receptor interior, and the lack of contacts near the intended interaction region. Among the poses that pass all of the filters, the pose with the highest DiffDock confidence score is selected as the initial structure for the subsequent MD simulations. The detailed filtering procedure and associated parameter settings are described in Supporting Information.

#### MD Simulations and MM/GBSA

CPP uptake and receptor-associated endocytic behavior are governed by multiple cellular processes and are therefore difficult to encode directly as objectives in a fully in silico optimization loop. We therefore used MD simulations followed by MM/GBSA analysis to assign each docked peptide–receptor complex a receptor-specific binding score. This score served as a computational proxy for receptor-associated interaction in the optimization loop. For peptide–receptor complex structures obtained from docking, preprocessing is performed using the protein preparation Wizard in the Schrödinger suite.^27^ Hydrogen bond networks are optimized, and protonation states of titratable residues are assigned at pH 7.0 using PROPKA.^32,37^ Terminal residues are capped as follows: ACE and NME groups are applied to the N- and C-termini of the receptor, respectively, while ACE and NH_2_ groups are applied to the CPP.

System topologies are generated using TLeap from AmberTools^4^ with the AMBER ff14SB force field.^28^ The complex is solvated using the TIP3P water model in a simulation box extending 12 Å beyond the outermost atoms of the complex. Na^+^ and Cl^–^ ions are added for charge neutralization, and the ionic strength is adjusted to 0.15 M. The resulting systems are converted to the GROMACS format using ACPYPE.^38^

MD simulations are performed using GROMACS.^1^ After energy minimization, the system was equilibrated for 100 ps under NVT while heating from 0 to 310 K, followed by 100 ps of equilibration under NPT at 310 K and 1 bar. A 100 ps production run was then performed under NPT at 310 K and 1 bar. A 2 fs timestep is used. Temperature and pressure were controlled using the V-rescale thermostat and the C-rescale barostat, respectively. Bonds involving hydrogen atoms are constrained by using LINCS. Snapshots were saved every 10 ps during the production run.

To evaluate peptide–receptor interaction strength in the optimization loop, a binding score is defined from the MM/GBSA binding free energy computed using gmx_MMPBSA.^42^ To reduce length-dependent bias across peptides of different lengths, the MM/GBSA value is normalized by the number of residues, and the sign is inverted so that larger values indicate stronger interaction:

1 $$Binding\;score=-\frac{\Delta G_{MM/GBSA}}{N_{res}}$$

For MM/GBSA calculations, the internal dielectric constant is set to 2, the external dielectric constant to 80, and the salt concentration to 0.15 M. The binding score defined in eq 1 and obtained using the 100 ps MD protocol was used as a receptor-wise computational screening proxy for rapid relative comparison of candidate sequences in the optimization loop.

#### Deep Ensemble Surrogate Model

To predict the receptor-wise binding scores and quantify their uncertainties for unevaluated sequences, a deep ensemble surrogate model is trained using the receptor-wise binding scores obtained as described above.^22^ Each model takes a CPP sequence as input, maps it to a hidden representation through an embedding layer, and processes the representation using one-dimensional convolutional layers. The output layer predicts the mean and variance of the binding scores for each receptor. Three architectures with different convolutional configurations are used, and four models are trained for each architecture with different random initializations, yielding a total of 12 models. Model training uses a negative log likelihood loss, treating the MM/GBSA binding score as a sample from a normal distribution. Model specifications and training details are provided in Supporting Information.

At each cycle, the deep ensemble is retrained using all binding score observations accumulated up to that point, and the mean and variance of the binding scores are predicted for the remaining un-evaluated sequences.

#### Multi-Objective Bayesian Optimization

To select sequences for MD simulations in the next cycle, multi-objective Bayesian optimization is applied using the deep ensemble surrogate model and qNEHVI (q Noisy Expected Hypervolume Improvement).^8^ qNEHVI seeks to expand the Pareto front by maximizing the expected hypervolume improvement while accounting for observation noise. This is appropriate in the present setting because the MM/GBSA-based scores vary owing to differences in initial structures and thermal fluctuations. In addition, qNEHVI allows for batch selection, which is suitable for large-scale parallel simulations. The reference point for qNEHVI is determined from the empirical distributions of the observed binding scores in the initial 10,000 evaluated sequences: μ – 2σ for the target receptor and μ + 2σ for the counter-objective receptor, where μ and σ denote the receptor-wise mean and standard deviation, respectively. In each cycle, qNEHVI is computed from the predictive distributions of the deep ensemble and used to select 2000 unevaluated sequences for MD simulations in the next cycle. Details of the acquisition calculation are provided in Supporting Information.

#### Selection of the Top Sequences

After the optimization loop, qNEHVI is computed using the trained deep ensemble while considering all of the simulated sequences as candidates. The top 10 sequences with the highest qNEHVI values were selected as candidates for experimental validation. For the sequence-level analyses, the top 100 sequences were defined as the 100 MD-evaluated sequences with the highest qNEHVI values in the same post-optimization ranking.

## Results

### Design of the CPP Exploration Space

To assess the effect of fine-tuning, we generated 1000 sequences of length 9–18 residues from the default EvoDiff model and 1000 from the fine-tuned model, and we computed the sequence embeddings using ESM2 (esm2_t33_650M_UR50D). We then defined a UMAP space using the embeddings from the default EvoDiff model and projected the default EvoDiff samples, fine-tuned samples, and known CPP sequences onto the same space (Figure 2a). Samples from the default EvoDiff model were broadly distributed across the embedding space, whereas fine-tuned samples were concentrated closer to the regions occupied by known CPPs.

**Figure 2:**
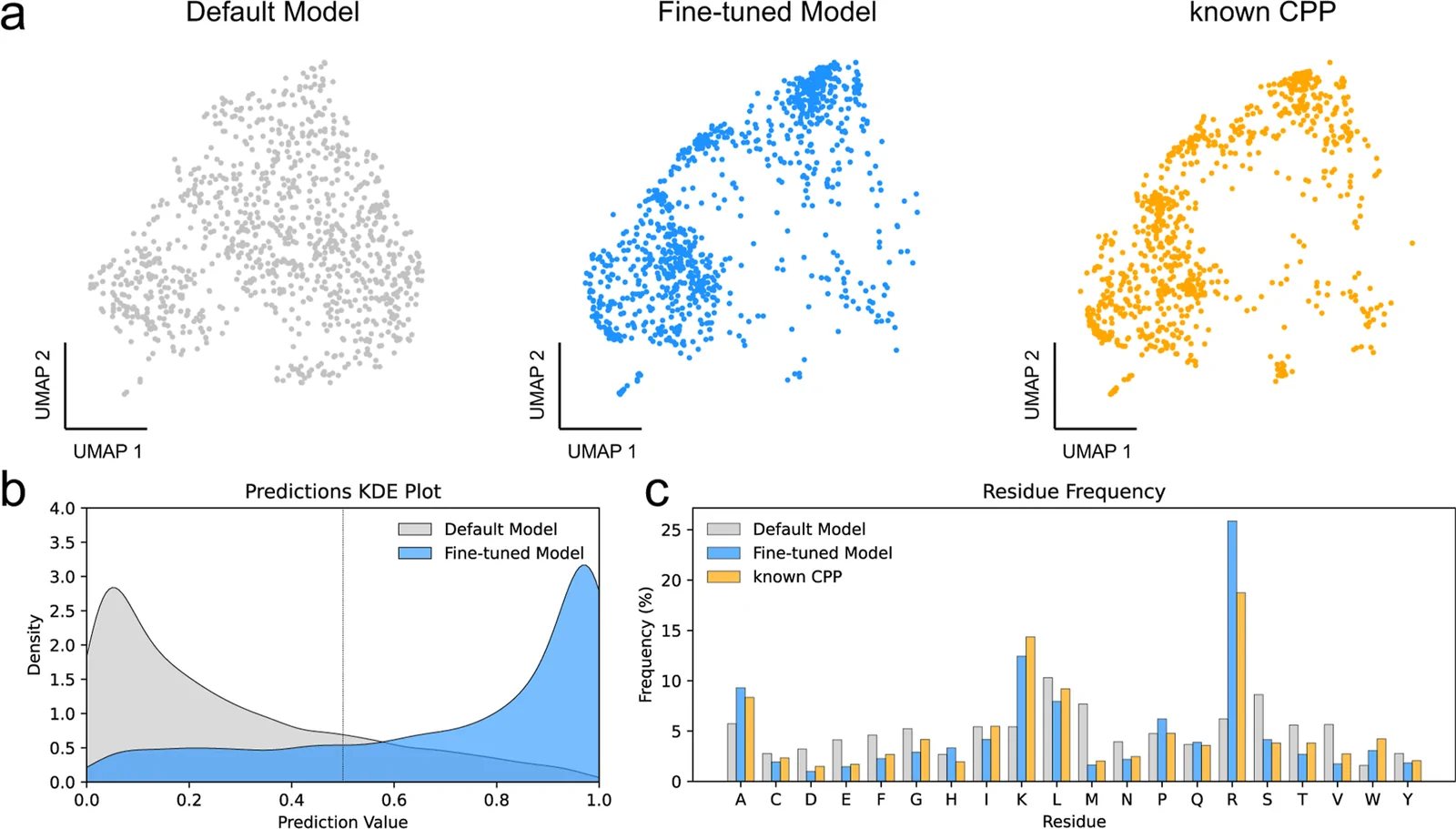
Construction of the CPP candidate library and sequence features before and after fine-tuning. (a) UMAP projection of ESM2 sequence embeddings. Left: default EvoDiff samples. Middle: fine-tuned samples. Right: known CPP sequences. (b) Kernel density estimation of CPP-likeness scores estimated by the trained CPP/non-CPP classifier for default EvoDiff samples and fine-tuned samples. (c) Amino acid residue frequency distributions for default EvoDiff samples, fine-tuned samples, and known CPPs.

We next evaluated CPP-likeness of the generated sequences using two approaches: a trained CPP/non-CPP classifier and the existing CPP prediction tool c3pred.^23^ The classifier was trained on data not used for fine-tuning; the data split, training procedure, and full performance metrics are described in Supporting Information. The classifier achieved an AUC of 0.9148 for an independent test set. We then computed the CPP-likeness scores for the generated sequences using both the classifier and c3pred and compared the score distributions between default EvoDiff samples and fine-tuned samples (Figures 2b and S3a,b). In both evaluations, fine-tuned samples shifted toward higher CPP-likeness than that of default EvoDiff samples.

We also compared the amino acid compositions between default EvoDiff samples and fine-tuned samples using known CPPs as a reference. Relative to the default EvoDiff model, the fine-tuned model generated sequences enriched in basic residues such as arginine and lysine while still retaining hydroxyl-containing and hydrophobic residues (Figure 2c). Pairwise Tanimoto distance distributions further indicated that the generated sequences retained sequence diversity (Figure S3c). These results indicate that fine-tuning shifted the generated sequence distribution toward known CPP-like features while preserving the sequence diversity. We therefore used the fine-tuned model to construct the candidate CPP library for a subsequent receptor-wise evaluation and optimization.

### Initial Receptor-Wise Binding Landscape

In this study, sequence design was formulated as a two-objective optimization problem in which CXCR4 was treated as the target receptor and NRP1 as the counter-objective receptor, with the aim of maximizing the CXCR4 binding score and minimizing the NRP1 binding score. We first defined the receptor-side interaction regions used for docking pose filtering and subsequent receptor-wise scoring. We used PDB ID 3ODU for CXCR4 and PDB ID 7JJC for NRP1 (Figure 3a). Since each optimization cycle required thousands of evaluations, the optimization loop used a computationally tractable 100 ps MD protocol. Details of this protocol and its assessment against longer-time scale simulations are provided in Supporting Information (S4).

**Figure 3:**
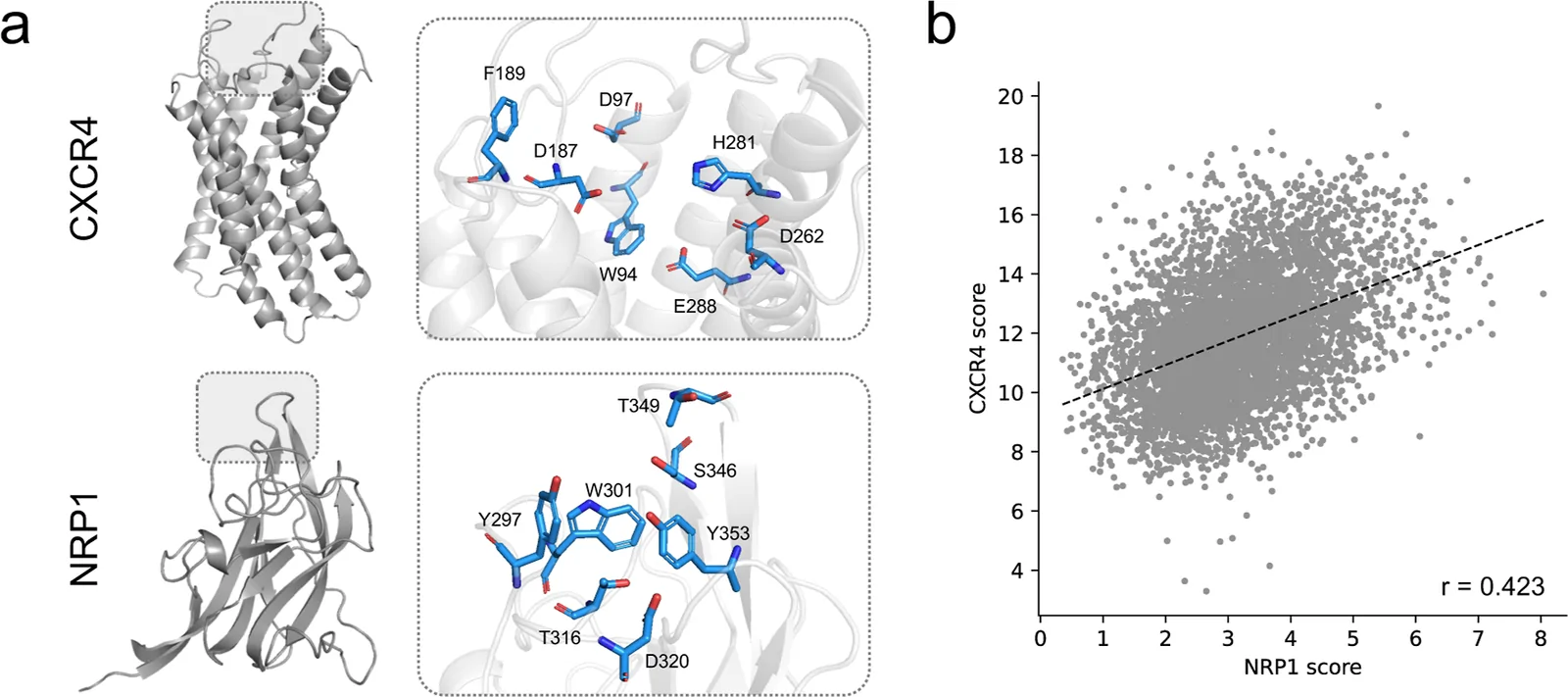
Docking target definition and correlation of receptor-wise binding scores. (a) Overall structures of the receptors. CXCR4 is shown on the left and NRP1 on the right. Receptor-side interaction regions used for docking-pose filtering are highlighted in blue. (b) Scatter plot of CXCR4 and NRP1 binding scores calculated for the initial candidate set of 10,000 sequences. The black dashed line indicates the linear regression line. Pearson’s correlation coefficient is *r* = 0.423 (*p* = 6.24 × 10^–226^).

Using this protocol, we docked the initial 10,000 candidate sequences against both receptors and computed receptor-wise binding scores by MD simulations and MM/GBSA for complexes that passed the pose filtering. Across the initial set, CXCR4 and NRP1 binding scores were positively correlated (Figure 3b), indicating that improvement in the CXCR4 binding score could unintendedly enhance the NRP1 binding score.

### Exploration of the Receptor-Selective CPP Candidates by Multi-Objective Bayesian Optimization

We performed multi-objective Bayesian optimization over the candidate CPP library using receptor-wise binding scores as objectives.

To monitor the surrogate model performance during optimization, we calculated the correlation between the predicted binding scores and the observed MM/GBSA binding scores on a validation subset. In each optimization cycle, the available evaluated sequences were randomly split into training and validation subsets at a ratio of 9:1, and the surrogate models were retrained on the training subset.

This correlation increased over the course of optimization (Figure 4a), indicating a progressive improvement in the agreement between predicted and observed scores.

**Figure 4:**
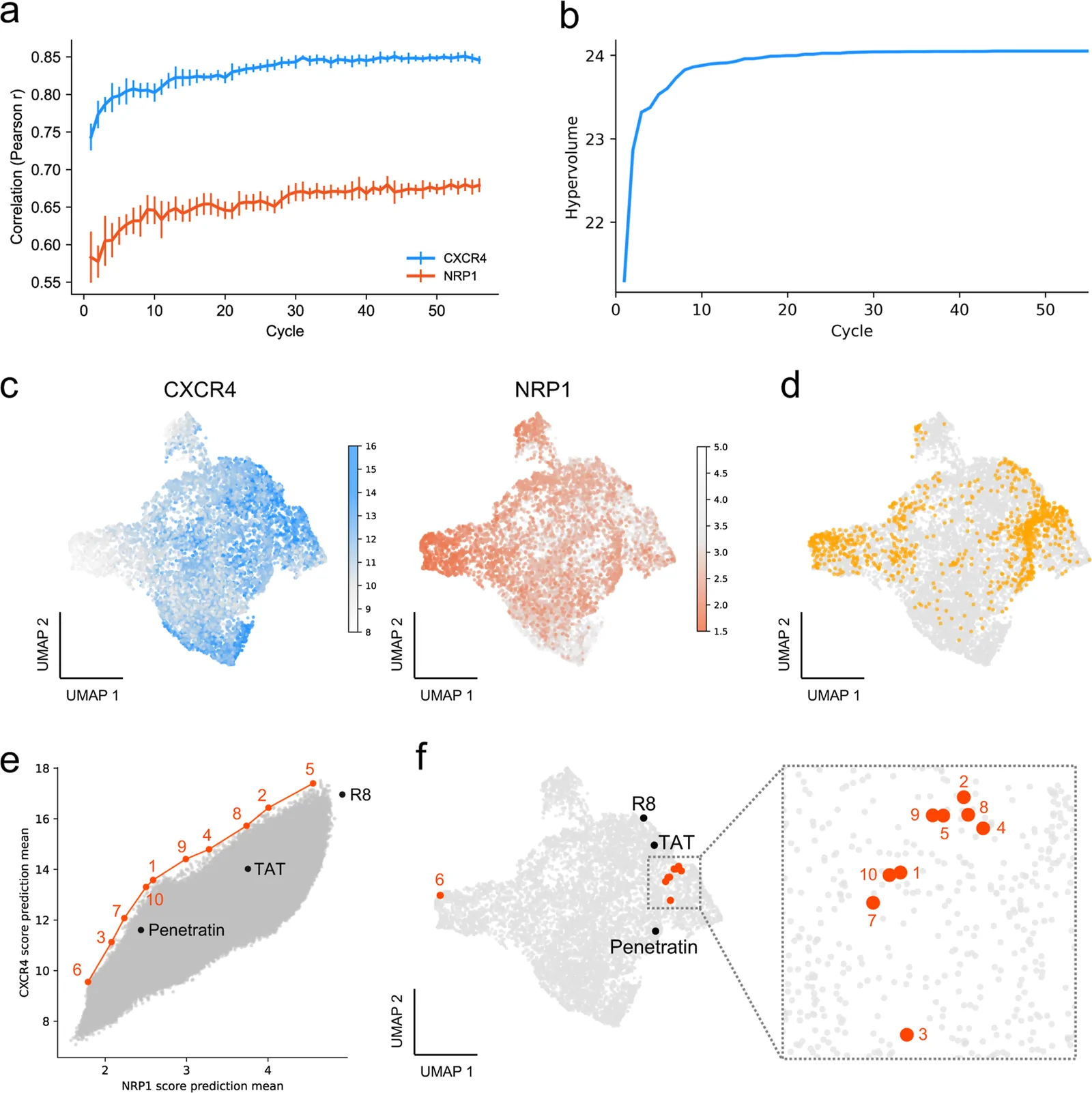
Exploration process and sequence distribution in multi-objective optimization. (a) Correlation coefficient between the deep ensemble mean predictions and the MM/GBSA binding scores on a validation subset in each optimization cycle. (b) Hypervolume values computed from predicted mean binding scores in each cycle using the deep ensemble model trained in the final cycle. (c) UMAP projection of ESM2 sequence embeddings colored by predicted binding scores. Left: CXCR4 predicted binding scores. Right: NRP1 predicted binding scores. (d) UMAP projection of the same embeddings highlighting sequences proposed by Bayesian optimization (yellow points). (e) Scatter plot of CXCR4 and NRP1 binding scores predicted by the surrogate model (gray points), with representative known CPPs and the selected sequences overlaid. The selected sequences are shown in red and numbered 1–10 (corresponding to S-01–S-10). (f) UMAP projection of all sequences, with the selected sequences shown in red and representative known CPPs shown in black.

We also performed cycle-wise prospective validation, in which surrogate models trained using data accumulated through the *N*-th cycle were evaluated on newly MM/GBSA-evaluated sequences from the subsequent (*N* + 1)-th cycle that were not included in the training data (Figure S5). Root mean squared error, coefficient of determination (*R* ^2^), and Spearman’s rank correlation coefficient (ρ) were calculated for each cycle. To quantify the optimization progress, we performed a post-hoc hypervolume analysis using predicted mean binding scores. Sequences obtained in each cycle were re-evaluated using the deep ensemble model trained in the final cycle, and the hypervolume was calculated relative to the reference point. Hypervolume increased in early cycles and then approached a plateau in later cycles (Figure 4b).

We also compared the qNEHVI-based selection with random selection from the same candidate library using the evaluated binding scores (Figure S6). qNEHVI-based selection accumulated peptides in the target region more rapidly than random selection while limiting accumulation in the inverse target region. This comparison indicates that the qNEHVI-guided loop selected candidates with the intended CXCR4/NRP1 score profile more efficiently than did random sampling.

To examine which regions of the sequence space were preferentially explored, we visualized surrogate-predicted binding scores on the ESM2/UMAP embedding space in the final cycle (Figure 4c) and overlaid the sequences proposed by Bayesian optimization on the same space (Figure 4d). The proposed sequences were concentrated in regions associated with higher predicted CXCR4 scores and lower predicted NRP1 scores.

After completing the optimization loop, we selected 10 top-ranked sequences, denoted S-01 to S-10, from the set of candidates evaluated by MD simulations. Table 1 summarizes their sequences, lengths, and predicted receptor-wise binding scores together with those of three representative known CPPs used for comparison in subsequent analysis: TAT, R8, and Penetratin.

**Table 1:** Amino Acid Sequences, Lengths, and Predicted Binding Scores for Representative Known CPPs (TAT, R8, Penetratin) and Selected Sequences (S-01 to S-10)

index	sequence	length	CXCR4 score	NRP1 score
TAT	YGRKKRRQRRR	11	14.02 ± 2.67	3.75 ± 1.03
R8	RRRRRRRR	8	16.96 ± 3.72	4.91 ± 1.56
Penetratin	RQIKIWFQNRRMKWKK	16	11.60 ± 2.20	2.44 ± 0.60
S-01	RWWWRWRWRWTWIWR	15	13.66 ± 3.15	2.68 ± 0.80
S-02	RRWRWRWRWK	10	16.34 ± 3.46	4.02 ± 1.33
S-03	KALWWWWFRHWWWKKKKK	18	11.18 ± 2.38	2.14 ± 0.58
S-04	WRWRWRWRWKKK	12	14.69 ± 3.02	3.28 ± 1.04
S-05	RWRWRWRWR	9	17.28 ± 3.72	4.56 ± 1.45
S-06	KLLKKLAKKWLKAALKLA	18	9.55 ± 1.36	1.84 ± 0.40
S-07	RWWWQWWWRWWWHWKKG	17	12.32 ± 2.91	2.33 ± 0.68
S-08	KRWRWRWRKK	10	15.67 ± 3.28	3.77 ± 1.24
S-09	WRWRWRWRWRWRWR	14	14.37 ± 3.22	3.04 ± 0.93
S-10	RWWSWWWWWWWRWWK	15	13.45 ± 3.13	2.61 ± 0.82

We then plotted the predicted CXCR4 and NRP1 scores for all candidate sequences and overlaid S-01 to S-10 together with representative known CPPs (Figure 4e). These sequences showed favorable predicted CXCR4/NRP1 score combinations consistent with the optimization objectives defined in this study. We also projected them onto the UMAP embedding relative to the full candidate library and to known CPPs (Figure 4f). We found that one of them, S-09 (WRWRWRWRWRWRWR), was present in the fine-tuning data set.

Furthermore, we evaluated sequence-level characteristics of the optimized S-01–S-10 sequences and the top 100 sequences using UMAP, edit-distance, and amino-acid frequency analyses (Figure S7). Edit-distance analysis confirmed that the top 100 sequences retained sequence-level diversity. Amino-acid frequency analysis revealed that, compared with the CPP-like candidate library, the top 100 sequences and S-01–S-10 showed a marked increase in Trp frequency, indicating that Trp-rich sequences were selected during optimization.

### Cell-Based Evaluation of Receptor-Associated Peptide Enrichment

To examine whether the selected sequences showed receptor-associated localization patterns consistent with the in silico predictions, we performed cell-based evaluation using confocal microscopy with fluorescently labeled peptides (TAT, R8, Penetratin, and selected peptides) and quantified the peptide enrichment relative to receptor-positive regions. HeLa cells were used as a tractable model system because both CXCR4 and NRP1 are expressed in HeLa cells and can be evaluated in the same cellular background under a common imaging workflow. Cells were incubated individually for 1 h with each fluorescently labeled peptide, followed by immunofluorescence staining for CXCR4 or NRP1.

To quantify receptor-associated localization, we analyzed the images using the procedure described in Supporting Information. Briefly, peptide-positive ROIs were defined from the peptide channel, receptor-positive ROIs were defined from the receptor channel, and an enrichment score was calculated as the ratio of the mean peptide intensity in receptor-positive ROIs to that in peptide-positive ROIs.

We first quantified and summarized the mean enrichment scores of each peptide with respect to CXCR4-positive and NRP1-positive regions (Figure 5a). TAT, R8, S-07, S-09, and S-10 were excluded from this comparison because the fluorescence intensity was insufficient for reliable quantification under the imaging conditions used. Among the quantified peptides, S-01, S-02, S-03, and S-08 showed higher enrichment scores with respect to CXCR4-positive regions than those of NRP1-positive regions. Also, Penetratin showed the same tendency.

**Figure 5:**
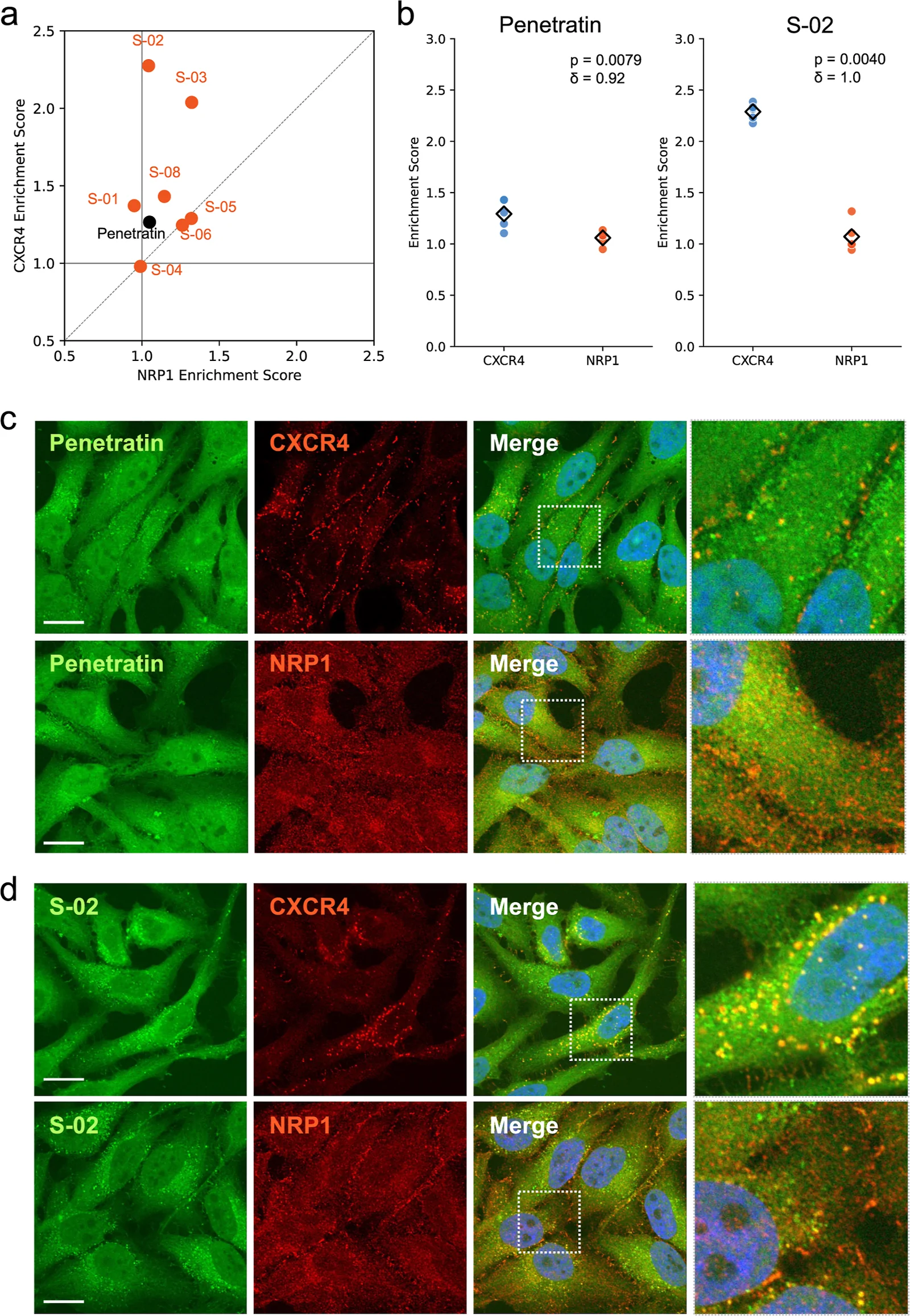
Cell-based evaluation of receptor-associated peptide enrichment. (a) Mean enrichment scores for quantified peptides with respect to CXCR4-positive and NRP1-positive regions. TAT, R8, S-07, S-09, and S-10 were excluded because fluorescence intensity was insufficient for reliable quantification. (b) Enrichment score distributions for Penetratin and S-02. *P* values were computed using a one-sided Mann–Whitney *U* test, and the corresponding effect sizes are indicated as Cliff’s δ. (c, d) Representative confocal images of Penetratin (c) and S-02 (d) under CXCR4 or NRP1 immunostaining. Green: peptide; red: receptor; blue: DAPI. Scale bar: 20 μm.

As representative examples from this analysis, the enrichment score distributions for Penetratin and S-02 are shown in Figure 5b. For both Penetratin and S-02, enrichment scores were significantly higher with respect to CXCR4-positive regions than NRP1-positive regions according to a one-sided Mann–Whitney *U* test, and the corresponding effect sizes are indicated as Cliff’s δ (Figure 5b). Representative confocal images showed punctate intracellular signals for both peptides, with Penetratin exhibiting partial overlap with CXCR4-positive regions and S-02 showing a qualitatively stronger tendency toward CXCR4-positive regions (Figure 5c,d). The enrichment score distributions for the other quantified peptides are provided in Figure S10.

These results indicate that several selected peptides showed higher enrichment in CXCR4-positive regions than in NRP1-positive regions under the tested conditions. To further characterize the S-02 enrichment pattern, we performed additional control experiments using free 5-FAM, scrambled peptides, and receptor knockout. Free 5-FAM alone did not produce clear intracellular fluorescence under the same imaging conditions (Figure S11). We next evaluated five scrambled peptides with the same amino acid composition as S-02. Predictions from the final-cycle surrogate model placed all five scrambled peptides near the predicted Pareto front, with CXCR4/NRP1 computational score profiles similar to those of S-02 (Figure S12a). In the imaging analysis, all five scrambled peptides retained a higher mean enrichment with respect to CXCR4-positive regions than NRP1-positive regions. However, four of the five scrambled peptides showed lower CXCR4-associated enrichment than S-02, whereas one showed a comparable level (Figure S12b,c). Following siRNA-mediated knockdown, the receptor fluorescence intensity was reduced for the corresponding receptor. CXCR4 knockdown decreased the S-02 enrichment score, whereas NRP1 knockdown increased it (Figure S13).

## Discussion

In this study, we developed a closed-loop multi-objective optimization framework for designing CPP candidates with receptor-wise interaction profiles. Specifically, we formulated the design problem as a multi-objective optimization over receptor-wise binding scores and solved it through a closed-loop in silico workflow. In the CXCR4/NRP1 case study, CXCR4 and NRP1 binding scores were positively correlated across the initial candidate set, indicating that sequences with stronger interactions with CXCR4 also tended to show stronger interactions with NRP1. This observation directly motivates the multi-objective formulation: a single-objective strategy that maximizes CXCR4 interaction alone would also tend to enrich peptides that interact strongly with NRP1, making receptor-selective design difficult to achieve. Despite this inherent trade-off, the optimization process identified candidate peptides with an improved predicted CXCR4-to-NRP1 score balance. Sequence-level analysis further showed that Trp-rich sequences became more frequent during optimization, while sequence diversity was retained among the top-ranked candidates (Figure S7). Furthermore, cell-based imaging provided supporting evidence that several of these candidates were enriched in CXCR4-positive regions to a greater extent than in the NRP1-positive regions. These findings support the value of formulating receptor-selective CPP design under competing receptor-interaction constraints as an explicit multi-objective optimization problem. Among the five composition-matched scrambled peptides located near the predicted Pareto front, four showed lower CXCR4-associated enrichment than S-02, whereas one showed a comparable level, suggesting that the residue arrangement may modulate the magnitude of enrichment (Figure S12). CXCR4 knockdown decreased S-02 enrichment, supporting a contribution of CXCR4 expression to the observed pattern, whereas NRP1 knockdown increased it (Figure S13). The latter response may partly reflect receptor cross-regulation, as NRP1 silencing has been reported to increase CXCR4 expression in HUVECs.^31^ Consistent with this possibility, CXCR4 immunofluorescence tended to increase under NRP1 knockdown conditions in the present experiment, although the underlying mechanism was not examined in this study. Thus, S-02 enrichment appears to include a CXCR4-associated component while also being influenced by a broader receptor expression context.

Three methodological aspects of this framework are particularly important in this context. First, the multi-objective formulation makes it possible to treat receptor-wise score trade-offs as explicit optimization objectives rather than as a property to be hoped for downstream; the output of optimization is therefore expected to be a set of candidates lying on the trade-off front between target and counter-objective receptor interactions, from which sequences biased toward the target side can be selected directly. Second, the receptor-wise binding score used as the optimization objective combines receptor-side pose filtering with length-normalized MM/GBSA, which allows peptides of different lengths and structurally distinct receptors to be compared on a common scale and reduces the risk that the optimizer would simply favor longer sequences or biologically implausible binding poses. Third, the closed-loop optimization architecture is particularly important for making broader exploration of CPP-like sequence space more practical. This architecture combines a CPP-like generative model,^2,16^ a deep ensemble surrogate,^22^ and a noise-aware multi-objective acquisition function (qNEHVI).^8^ Rather than restricting design to the local sequence changes typical of grafting-style or motif-based strategies,^11,25,29,30^ the framework concentrates computationally expensive receptor-wise evaluation on candidates most likely to improve the multi-objective trade-off, without requiring exhaustive screening.

The potential utility of this framework is not limited to the CXCR4/NRP1 case examined here. In principle, other receptor-selective design settings can also be addressed within the same framework. This is because neither the receptor-wise scoring procedure nor the closed-loop optimization is specific to a particular receptor structure; only the receptor pair and the associated optimization objectives need to be redefined. Moreover, the number of objectives is not restricted to two. Cytotoxicity and aggregation propensity, for example, are both relevant constraints in the development of peptide-based drug delivery systems. CPPs can show sequence- and cargo-dependent cytotoxicity through strengthened nonspecific interactions with cellular membranes,^9,21^ and aggregation is a well-recognized developability challenge for peptide and protein therapeutics.^43^ In principle, cytotoxicity could be incorporated using peptide-toxicity predictors or task-specific models trained on annotated data sets, whereas aggregation propensity could be incorporated using sequence-based aggregation predictors. Extending the framework in this way would add further objectives to the existing multi-objective formulation without requiring changes to the underlying optimization architecture and could enable the search for candidates that balance receptor-associated interaction profiles with practical developability requirements.

A central limitation of the present study is that the framework was optimized against computational receptor-wise scores rather than against biological selectivity itself. The current framework operates on a computational proxy: the multi-objective formulation treats receptor-wise scores as the design objective, the binding score itself is a physics-based approximation derived from a short molecular dynamics simulation followed by MM/GBSA, and the closed-loop optimization explores the trade-off front defined by these computed scores rather than the trade-off front defined by measured cellular phenotypes. Receptor-associated enrichment in cell-based imaging provides supporting evidence that the identified peptides captured biologically relevant differences, but this readout should not be regarded as a complete measure of the selectivity. In practice, biological selectivity is also influenced by processes beyond receptor interaction strength and local enrichment, including membrane perturbation, endocytic routing, intracellular trafficking, and endosomal escape.^10,14,36,40^ A natural next step is therefore to incorporate experimental readouts such as cellular uptake, intracellular localization, or receptor-associated enrichment directly into the design loop combined with the computational proxy. This would consider not only optimizing computationally receptor-wise proxies but also optimizing experimentally observed cellular phenotypes. The present study establishes a starting point for moving receptor-selective CPP design from in silico optimization based on proxy objectives toward closed-loop optimization of experimentally defined cellular functions.

## Supplementary Material



## Data Availability

The fine-tuning code and trained weights for the sequence generative model, the optimization-cycle pipeline code, and the Fiji macro used for image quantification are available on GitHub (https://github.com/IoriYamahata/CPP_Design). Note that the provided source code is a simplified version intended for concept verification; specific implementations for large-scale parallel computing (multi-node execution) used in the supercomputing environment have been omitted to ensure portability. All other data supporting the findings of this study are included in the main text and the Supporting Information.

## Notes

Additional computational and experimental methods; supplementary validation analyses for the MD protocol, surrogate model, optimization baseline, and optimized sequences; confocal imaging controls and enrichment-score analyses; and deep ensemble and image-processing parameters (PDF)
