# Supplementary material for: Closed-Loop Multi-Objective Optimization for Receptor-Selective Cell-Penetrating Peptide Design
Source: J Chem Inf Model. 2026 Sep 10;66(18):11642–53. doi: 10.1021/acs.jcim.6c01267 (PMC13625959; doi:10.1021/acs.jcim.6c01267)
Supplement: Supplementary file 1 [file ci6c01267_si_001.pdf]

Supporting Information

Closed-Loop Multi-Objective Optimization for Receptor-Selective Cell-Penetrating Peptide Design

Iori Yamahata*[1,2], Hidetomo Yokoo[3], Yosuke Demizu[3,4,5], Teppei Shimamura*[2], and Shuto Hayashi*[2,6]

[1] Laboratory for Data-driven Biology, Nagoya University Graduate School of Medicine, 65 Tsurumai-cho, Showa-ku, Nagoya, Aichi 466-8550, Japan
[2] Department of Computational and Systems Biology, Division of Biological Data Science, Medical Research Laboratory, Institute of Integrated Research, Institute of Science Tokyo, 1-5-45 Yushima, Bunkyo-ku, Tokyo 113-8510, Japan
[3] Division of Organic Chemistry, National Institute of Health Sciences, 3-25-26 Tonomachi, Kawasaki-ku, Kawasaki, Kanagawa 210-9501, Japan
[4] Graduate School of Medical Life Science, Yokohama City University, 1-7-29 Suehiro-cho, Tsurumi-ku, Yokohama, Kanagawa 230-0045, Japan
[5] Graduate School of Medicine, Dentistry and Pharmaceutical Sciences, Okayama University, 1-1-1 Tsushima-naka, Kita-ku, Okayama, Okayama 700-8530, Japan
[6] Innovation Center of NanoMedicine, Kawasaki Institute of Industrial Promotion, 3-25-14 Tonomachi, Kawasaki-ku, Kawasaki, Kanagawa 210-0821, Japan

Fine-tuning of the Sequence Generative Model. To adapt EvoDiff OADM640M to the CPP sequence distribution, EvoDiff is fine-tuned on experimentally reported CPP sequences using Low-Rank Adaptation (LoRA), in which trainable low-rank matrices are introduced while the pretrained model weights are kept fixed.
In this study, LoRA is applied to the `MaskedConv1d` layers in EvoDiff, which capture local sequence features through one-dimensional convolutions. The convolution kernel size is 5, and the LoRA hyperparameters are set to $r = 8$, $\alpha = 2$, and dropout $= 0.0$.
Fine-tuning is performed on the 1,082 CPP sequences collected from CellPPD, as described in the main text. All sequences are converted to uppercase amino-acid strings, and the dataset is randomly split into training and validation sets in a 9:1 ratio. Training uses the `OAMaskedCrossEntropyLoss` implemented in EvoDiff and the Adam optimizer with a weight decay of 0. An exponential learning-rate schedule is applied using a LambdaLR scheduler: the learning rate is increased exponentially from 1×10^{-7} to 1×10^{-4} during the first 10% of epochs, held at 1×10^{-4} until 50% of epochs, and then decayed exponentially to 1×10^{-7} over the remaining epochs. Fine-tuning is run for 3,000 epochs with a batch size of 100, and the final adapted model is used for sequence generation.

Training of the CPP Classifier. To assess whether the fine-tuned generative model produces sequences with CPP-like characteristics, we train a binary classifier to distinguish CPPs from non-CPPs using the dataset reported by Park et al. [1]. The development dataset consists of 561 CPP sequences and 1,097 non-CPP sequences, none of which are used for fine-tuning the generative model. These sequences are split into training and validation sets in an 8:2 ratio by stratified random sampling based on the CPP/non-CPP labels, using a fixed random seed. For final evaluation, we use the independent test set provided by Park et al., which consists of 150 CPP and 150 non-CPP sequences.
Input sequences are integer-encoded using a 20-amino-acid vocabulary with an additional PAD token. Sequences are right-padded, and the maximum sequence length is set to the maximum length observed in the development dataset. The classifier consists of an embedding layer, two one-dimensional convolutional layers with kernel sizes of 3 and 5, each followed by batch normalization and dropout, and a fully connected output layer. The embedding dimension and hidden dimension are both set to 16, and the dropout rate is 0.2.
The model is trained for 1,000 epochs with a batch size of 16 using binary cross-entropy loss with logits and the Adam optimizer with a learning rate of 1×10^{-5}. The model checkpoint with the lowest validation loss is selected for final evaluation. On the independent test set, each sequence is classified as CPP when the predicted probability exceeds 0.5 and as non-CPP otherwise. The classifier achieved an AUC of 0.9148, an accuracy of 84.00%, a precision of 0.8643, a recall of 0.8067, and an F1 score of 0.8345. The trained classifier is then applied to the generated sequences to estimate the probability that each sequence is CPP-like. The architecture of the classifier is shown in Fig. S1.

Selection of Docking Poses. To define an initial structure for MD simulation from the multiple poses generated by DiffDock, 20 docking poses are prepared for each peptide-receptor pair and filtered using the criteria below.
(1) Exclusion of poses with intrapeptide clashes.
If at least one pair of non-covalently bonded atoms in the peptide has an interatomic distance of 1.5 Å or less, the pose is considered to contain an intrapeptide clash and is excluded.
(2) Exclusion of poses with peptide penetration into the receptor interior.
To detect poses in which the peptide unnaturally penetrates into the receptor interior, we use the S function defined in

Eq. S1:

$$S(\mathbf{x}) = -\rho \ln \left(\sum_{j \in I_{\text{pro}}} \exp \left(-\frac{\|\mathbf{x} - \mathbf{x}_j\|^2}{\rho} \right) \right), \tag{S1}$$

where $\mathbf{x}$ denotes the 3D coordinates of a peptide atom, $\mathbf{x}_j$ denotes the 3D coordinates of the j-th receptor atom, and I_{pro} denotes the index set of all receptor atoms. A pose is excluded if at least one peptide atom satisfies

$$\min_{\mathbf{x} \in \text{peptide atoms}} S(\mathbf{x}) < \gamma.$$

The hyperparameters are set to $\rho = 0.3$ and $\gamma = 0.1$, respectively.

(3) Exclusion of poses lacking contacts near the intended interaction region.

To exclude poses located far from the receptor-side interaction region of interest, receptor-specific key residues are defined, and at least one peptide atom is required to contact this region. A pose is excluded if no peptide atom is within 4.2 Å of any atom in the corresponding receptor-specific key residues. For CXCR4, the key residues are W94, D97, D187, F189, D262, H281, and E288 based on prior structural and functional analyses of the receptor [2]. For NRP1, the key residues are Y297, W301, T316, D320, S346, T349, and Y353 based on structural analysis of the CendR binding pocket [3].

Among the poses that pass all three filters, the one with the highest DiffDock confidence score is selected as the initial structure for subsequent MD simulations. If no pose passes all filters for a given peptide-receptor pair, that pair is excluded from subsequent MD simulations.

Validation of the 100 ps MD Protocol. Since each optimization cycle requires thousands of evaluations, the main optimization loop employed a computationally tractable 100 ps MD protocol as a screening proxy for rapidly comparing candidate peptides. To assess whether this short-timescale protocol retained the sequence-dependent score trends required for such relative comparisons, we compared the 100 ps condition with a longer 100 ns reference condition using the 10 peptides selected from candidates evaluated during the optimization loop (S-01 to S-10), each evaluated against both CXCR4 and NRP1. For each peptide-receptor pair, 10 simulations were initiated from 10 different initial structures generated by DiffDock. The 100 ps simulations were performed using the same protocol as that adopted in the main optimization loop.

For the 100 ns reference condition, CXCR4, a transmembrane receptor, was simulated in a membrane-embedded system, whereas NRP1, treated as an ectodomain receptor, was simulated in aqueous solution without an explicit membrane. For CXCR4, the membrane orientation and position relative to the protein were estimated using the OPM/PPM server [4, 5], and a bilayer composed of 30% cholesterol and 70% POPC was constructed using the Membrane Builder in CHARMM-GUI [6, 7]. The force field, water model, ion concentration, and other simulation settings were the same as those used in the main MD protocol. Binding scores for the 100 ns simulations were calculated by MM/GBSA analysis using 100 frames sampled at intervals of 1,000 frames from each trajectory. Simulations with sign-inverted MM/GBSA-derived energy values greater than 100 before normalization by the number of residues were regarded as crashed trajectories and were excluded from subsequent analysis. For both timescales, MM/GBSA scores were averaged across the initial structures to obtain one score for each peptide-receptor pair, and Pearson and Spearman correlations between the 100 ps and 100 ns scores were calculated for each receptor.

Deep Ensemble Model Architecture and Training. To predict receptor-wise binding scores from peptide sequences during the optimization loop, deep ensemble surrogate models are trained. The models take peptide sequences as input and output the mean and variance of the predicted binding score for each receptor. The architecture of the surrogate model is illustrated in Fig. S2. Input sequences are encoded as integer amino-acid indices using the amino-acid dictionary. They are then right-padded with the PAD token to the maximum sequence length defined in the model configuration, and sequences longer than this length are truncated. The encoded sequence is mapped to a hidden representation through an embedding layer and then processed by repeated one-dimensional convolutional blocks to extract local sequence features. These features are flattened into a fixed-length representation and passed to regression heads that output the mean and variance of the binding score for each receptor, with the variance output constrained to be positive using the softplus function.

Using this basic structure, three model configurations with different convolutional settings are employed. For each configuration, four models are trained with different random initializations, yielding 12 models in total. Architecture-specific hyperparameters are summarized in Table S1.

Before training, sequences are excluded if any receptor-wise sign-inverted MM/GBSA-derived energy value exceeds a predefined threshold before normalization by the number of residues. Such cases are regarded as crashed trajectories. In each optimization cycle, the available dataset is randomly split into training and validation subsets at a 9:1 ratio. All models are trained with a batch size of 500 and a maximum sequence length of 18 using Gaussian negative log-likelihood loss and the Adam optimizer with a weight decay of 1×10^{-5}. Training is performed for up to 5,000

epochs. The checkpoint with the lowest validation loss is retained, and early stopping is applied based on validation loss using the patience value specified for each model configuration. The predictive mean and variance used in downstream acquisition are obtained by aggregating the outputs of the 12 ensemble members.

As a cycle-wise prospective validation, the 12 surrogate models trained using data accumulated through the N-th optimization cycle were evaluated on newly MM/GBSA-evaluated sequences from the subsequent $(N+1)$-th cycle that were not included in the training data. Root mean squared error (RMSE), coefficient of determination (R^2), and Spearman's rank correlation coefficient (ρ) were calculated separately for each ensemble member.

Details of Multi-objective Bayesian Optimization. To select peptide sequences for MD simulation in each optimization cycle, we use a deep-ensemble-based qNEHVI acquisition function [8]. Since qNEHVI is formulated for maximization, the two-objective vector for each sequence x is defined as

$$\mathbf{f}(x) = \left(f_{\text{target}}(x), -f_{\text{counter}}(x)\right),$$

where $f_{\text{target}}(x)$ and $f_{\text{counter}}(x)$ denote the actual binding scores for the target and counter-objective receptors, respectively.

Let $M = 12$ denote the number of surrogate models in the deep ensemble. For each surrogate model m, receptor-wise predictive means are computed for both previously evaluated sequences and unevaluated candidate sequences. The model-wise predictive mean vector for sequence x is defined as

$$\boldsymbol{\mu}_m(x) = \left(\mu_{m,\text{target}}(x), -\mu_{m,\text{counter}}(x)\right), \qquad m = 1, \ldots, M.$$

In the acquisition calculation, the model-wise predictive mean vectors are treated as ensemble-derived samples of the objective landscape. Accordingly, for each surrogate model m, a model-specific Pareto set is constructed from the predicted objective vectors of the previously evaluated sequences,

$$P_m = \text{Pareto}\left(\{\boldsymbol{\mu}_m(x) : x \in X_{\text{obs}}\}\right),$$

where X_{obs} denotes the set of previously evaluated sequences. The variance outputs of the surrogate models are not used in the acquisition calculation.

For a candidate batch X_{cand}, the model-specific hypervolume improvement is defined as

$$\text{HVI}_m(X_{\text{cand}}) = \text{HV}(P_m \cup \{\boldsymbol{\mu}_m(x) : x \in X_{\text{cand}}\}; \mathbf{r}) - \text{HV}(P_m; \mathbf{r}),$$

where $\mathbf{r}$ denotes the reference point and $\text{HV}(\cdot; \mathbf{r})$ denotes the hypervolume measured with respect to $\mathbf{r}$. The acquisition value is defined as the expectation of the model-specific hypervolume improvement under the empirical distribution over ensemble members:

$$\hat{\alpha}_{q\text{NEHVI}}(X_{\text{cand}}) = \frac{1}{M} \sum_{m=1}^{M} \text{HVI}_m(X_{\text{cand}}).$$

The reference point $\mathbf{r}$ is defined from the empirical distributions of the observed binding scores in the initial 10,000 evaluated sequences. Specifically, the reference point is set to $\mu - 2\sigma$ for the target receptor and $\mu + 2\sigma$ for the counter-objective receptor, where μ and σ denote the receptor-specific mean and standard deviation, respectively. In each cycle, acquisition values are computed for previously unevaluated sequences, and the top 2,000 sequences are selected for MD simulation in the next cycle.

Random-selection baseline for optimization analysis. To evaluate the selection efficiency of qNEHVI-based exploration, we constructed a random-selection baseline by sampling peptides from the same CPP-like candidate library using the same batch size as the optimization loop. The thresholds for region assignment were defined from the binding-score distributions obtained across the full random-selection baseline. The target region was defined as the region in which the CXCR4 binding score was greater than or equal to the top 10% threshold of the random baseline and the NRP1 binding score was less than or equal to the bottom 10% threshold. Conversely, the inverse target region was defined as the region in which the CXCR4 binding score was less than or equal to the bottom 10% threshold and the NRP1 binding score was greater than or equal to the top 10% threshold. For each optimization cycle, we calculated the cumulative percentage of selected peptides located in the target or inverse target region using the evaluated binding scores, and compared the qNEHVI-based exploration with the random-selection baseline (Fig. S6).

Peptide Synthesis. Candidate peptide sequences were chemically synthesized by Scrum Inc. (Tokyo, Japan). To enable fluorescence visualization, the N-terminus of each peptide was labeled with 5-carboxyfluorescein (5-FAM), and the C-terminus was amidated. The purity of all synthesized peptides was confirmed to be greater than 95% by high-performance liquid chromatography (HPLC), and the molecular weight of each peptide was verified by mass spectrometry (MS).
For the scrambled peptide analysis, five scrambled variants of S-02 with the same amino acid composition were synthesized, labeled with 5-FAM at the N-terminus, and amidated at the C-terminus. The peptides were purified by reversed-phase HPLC using 0.1% TFA in water and 0.1% TFA in acetonitrile as the mobile phases and characterized by analytical HPLC and MS.

Confocal Microscopy. HeLa cells were maintained in DMEM supplemented with 10% fetal bovine serum (FBS) at 37°C in a humidified atmosphere containing 5% CO_2. For confocal microscopy, cells were seeded at 5×10^4 cells/well in 24-well plates containing 13-mm round coverslips. Peptides labeled with 5-FAM were added to the culture medium at a final concentration of $10\ \mu\mathrm{M}$ in a total volume of $500\ \mu\mathrm{L}$ per well, and the cells were incubated for 1 hour at 37°C. After incubation, cells were fixed with 4% paraformaldehyde in PBS for 15 min at room temperature, permeabilized with 0.2% Triton X-100 in PBS for 15 min at room temperature, and blocked with 2% BSA in PBS for 30 min at room temperature. Cells were then stained for 1 hour at room temperature with either a rabbit anti-CXCR4 primary antibody (ab124824, Abcam) or a rabbit anti-NRP1 primary antibody (ab81321, Abcam), each diluted 1:1,000, followed by incubation for 1 hour at room temperature with a goat anti-rabbit IgG (H+L), cross-adsorbed, Alexa Fluor 594-conjugated secondary antibody (A-11012, Invitrogen) diluted 1:1,000. Cells were washed three times with PBS after fixation, permeabilization, blocking, and each antibody incubation step. Nuclei were counterstained using ProLong Gold Antifade Mountant with DAPI (P10144, Invitrogen).
Images were acquired using an EVIDENT FV4000 confocal laser scanning microscope with a UPLXAPO60XO 60×/1.42 NA objective lens. DAPI, 5-FAM, and Alexa Fluor 594 signals were acquired using the 405-, 488-, and 561-nm laser lines, respectively.

Free-fluorophore and scrambled-peptide controls. For the free-fluorophore control, free 5-FAM was used at a final concentration of $10\ \mu\mathrm{M}$ under the same imaging conditions as 5-FAM-labeled S-02. For the scrambled-peptide analysis, S-02 and five scrambled variants with the same amino-acid composition were used: S-02, 5-FAM-RRWRWRWRWK-NH_2; scr-1, 5-FAM-RRRRKRWWWW-NH_2; scr-2, 5-FAM-RRRRRWKWWW-NH_2; scr-3, 5-FAM-WWRRKRRWWR-NH_2; scr-4, 5-FAM-WWRRRWRKWR-NH_2; and scr-5, 5-FAM-WWWWRRRKRR-NH_2. Each peptide was used at a final concentration of $10\ \mu\mathrm{M}$ and analyzed using the same confocal microscopy and enrichment-score quantification procedures as described above.

siRNA-mediated receptor knockdown. For receptor knockdown analysis, siRNAs were introduced using the Neon Transfection System. Silencer Select siRNAs targeting human CXCR4 (s15412 and s15413; Thermo Fisher Scientific), Silencer Select siRNAs targeting human NRP1 (s16843 and s16844; Thermo Fisher Scientific), and Silencer Select Negative Control No. 1 siRNA (4390843; Thermo Fisher Scientific) were used. Electroporation was performed at 1005 V, 35 ms, and 2 pulses, with a cell density of 5×10^6 cells/mL and a final siRNA concentration of 100 nM. At 48 h after electroporation, S-02 imaging and receptor immunostaining were performed using the confocal microscopy procedure described above.

Quantification of Peptide Enrichment. Quantitative image analysis was performed in Fiji using a custom macro script. The main image-processing parameters are summarized in Table S2. The mean peptide fluorescence intensity was measured within the receptor-positive region (I_{Receptor}) and within the peptide-positive region (I_{Peptide}). The enrichment score was defined as

$$ES = \frac{I_{\mathrm{Receptor}}}{I_{\mathrm{Peptide}}}.$$

The final enrichment score for each image was obtained by averaging the four quadrant-level values. Full implementation details are provided in the Fiji macro available in the GitHub repository listed in the Data availability section. Representative raw images together with the corresponding peptide-positive and receptor-positive masks for Penetratin and S-02 are shown in Figs. S8 and S9, respectively.

Supplementary Figures

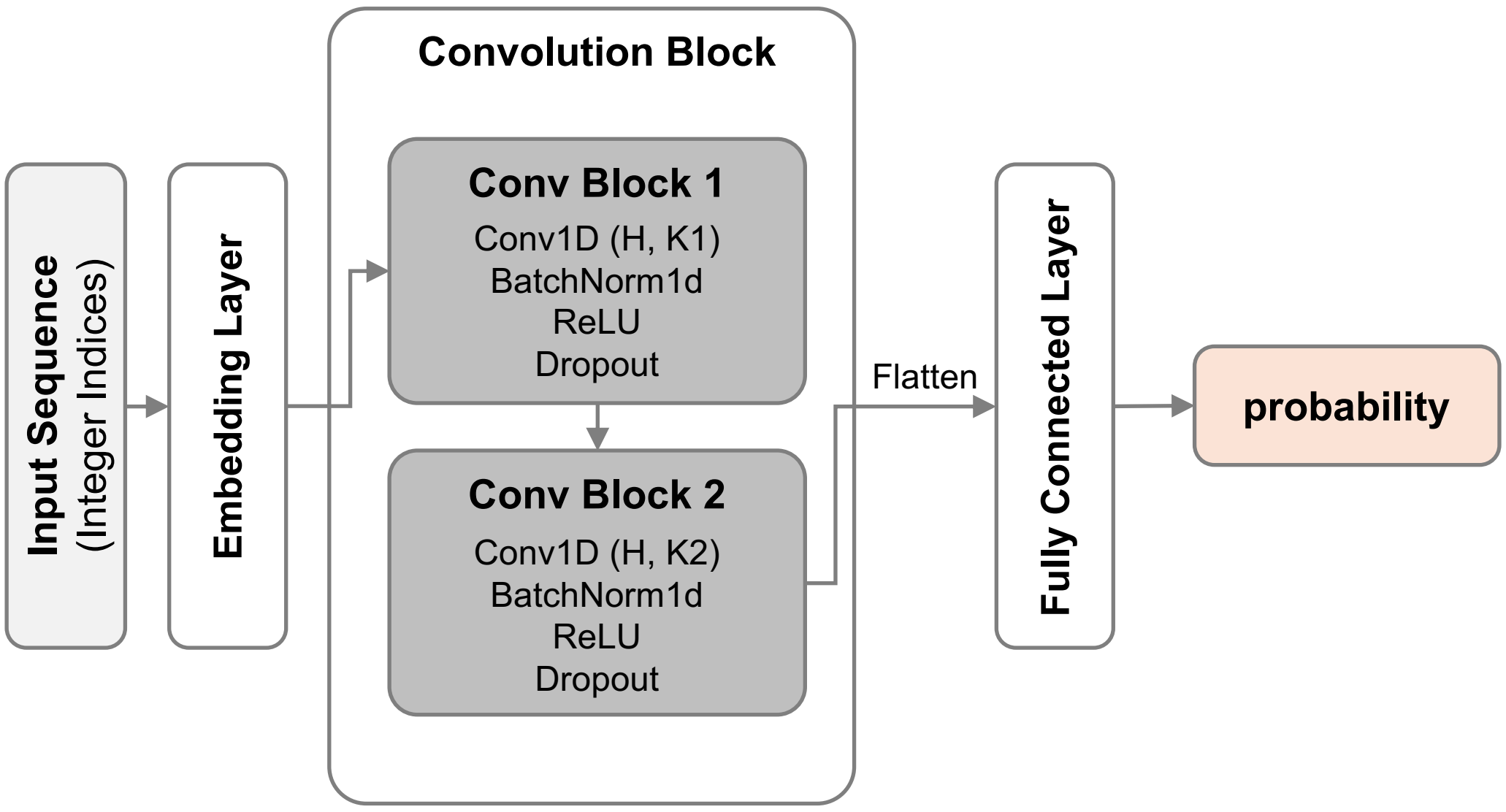

Figure S1. Architecture of the CPP binary classifier used in this study.
Schematic illustration of the 1D-CNN model used to evaluate whether the generated sequences possess CPP-like characteristics. Input sequences were integer-encoded using a 20-amino-acid vocabulary with an additional PAD token, right-padded to the maximum sequence length observed in the development dataset, and passed through an embedding layer. The model consists of two 1D convolutional layers followed by batch normalization and dropout, a flattening layer, and a fully connected output layer followed by a sigmoid activation. Here, H denotes the number of convolutional output channels, and K1 and K2 denote the kernel sizes of the first and second convolutional layers, respectively. In this classifier, the embedding dimension and hidden dimension were both set to 16, the kernel sizes were 3 and 5, the dilation factor was fixed to 1 for both layers, and the dropout rate was 0.2.

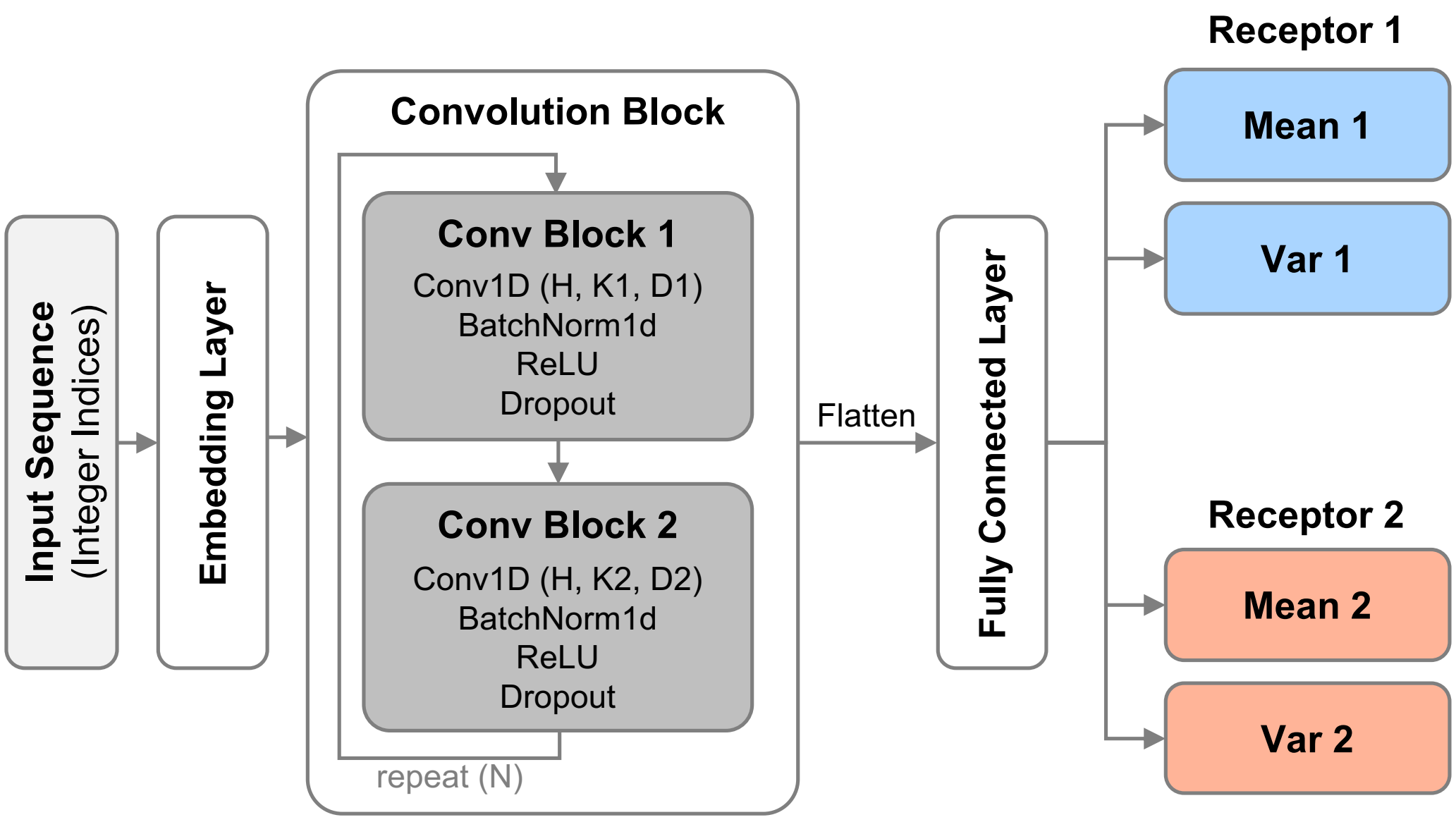

Figure S2. Architecture of the deep ensemble surrogate model.
Schematic illustration of the convolutional regression model used to predict receptor-wise binding scores. For each receptor, the model outputs the predicted mean (μ) and variance (σ^2) of the binding score. Convolutional layers extract local sequence patterns, which are then flattened and passed to regression heads that generate these outputs. Here, H denotes the hidden dimension (number of convolutional output channels), K1 and K2 denote the kernel sizes of the first and second convolutional layers, and D1 and D2 denote the corresponding dilation factors. The figure shows the common model structure shared across the ensemble, while architecture-specific hyperparameter settings are summarized in Table S1.

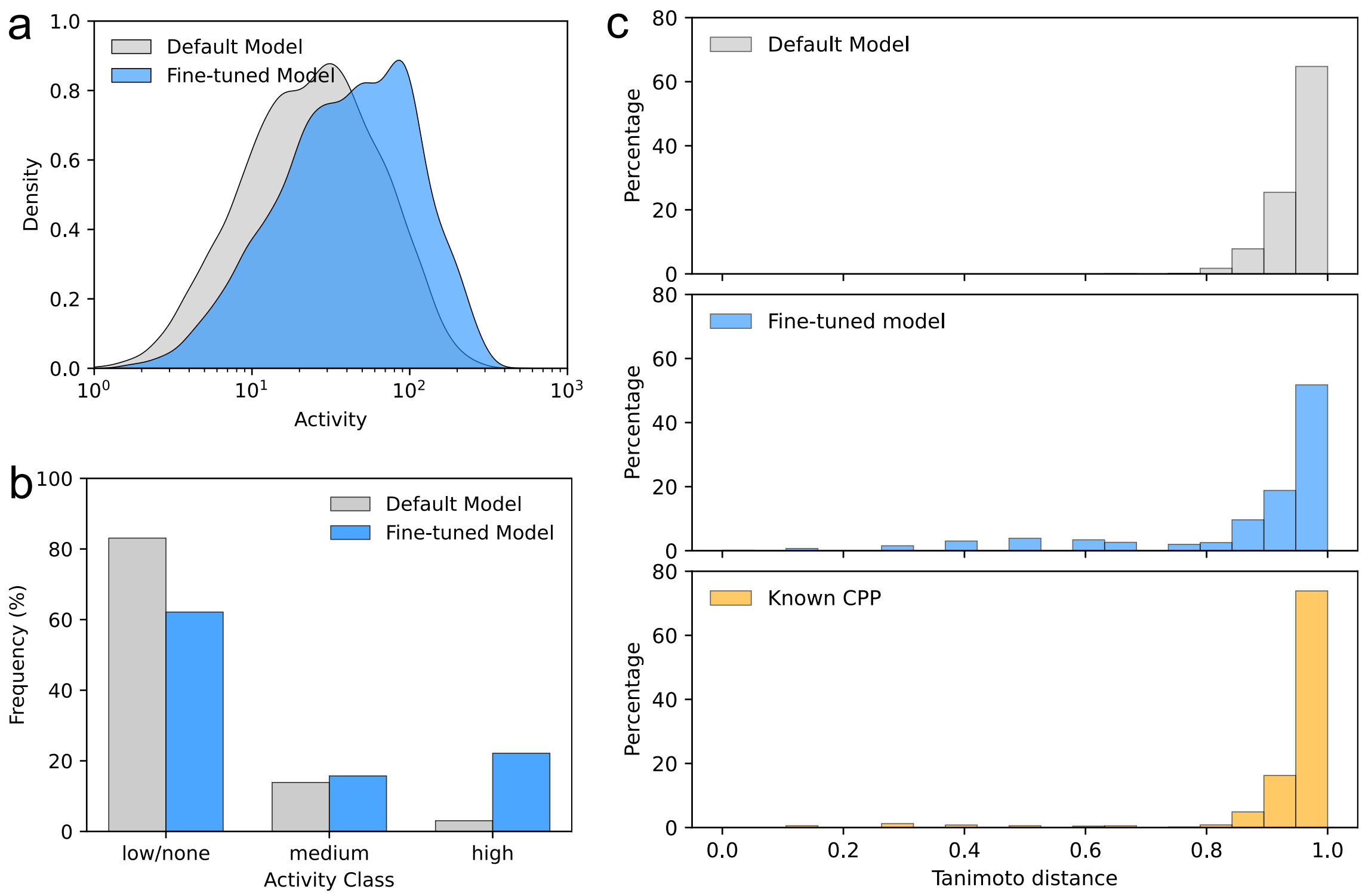

Figure S3. Evaluation of generated sequence characteristics.
(a) Distribution of continuous CPP activity scores predicted by c3pred. (b) Activity class predictions by c3pred. (c) Histograms showing the distribution of pairwise Tanimoto distances for sequences, calculated on binary sequence fingerprints constructed by concatenating position-wise one-hot encodings of amino-acid identities. Top: sequences from the default EvoDiff model. Middle: sequences from the fine-tuned model. Bottom: known CPP sequences (training data).

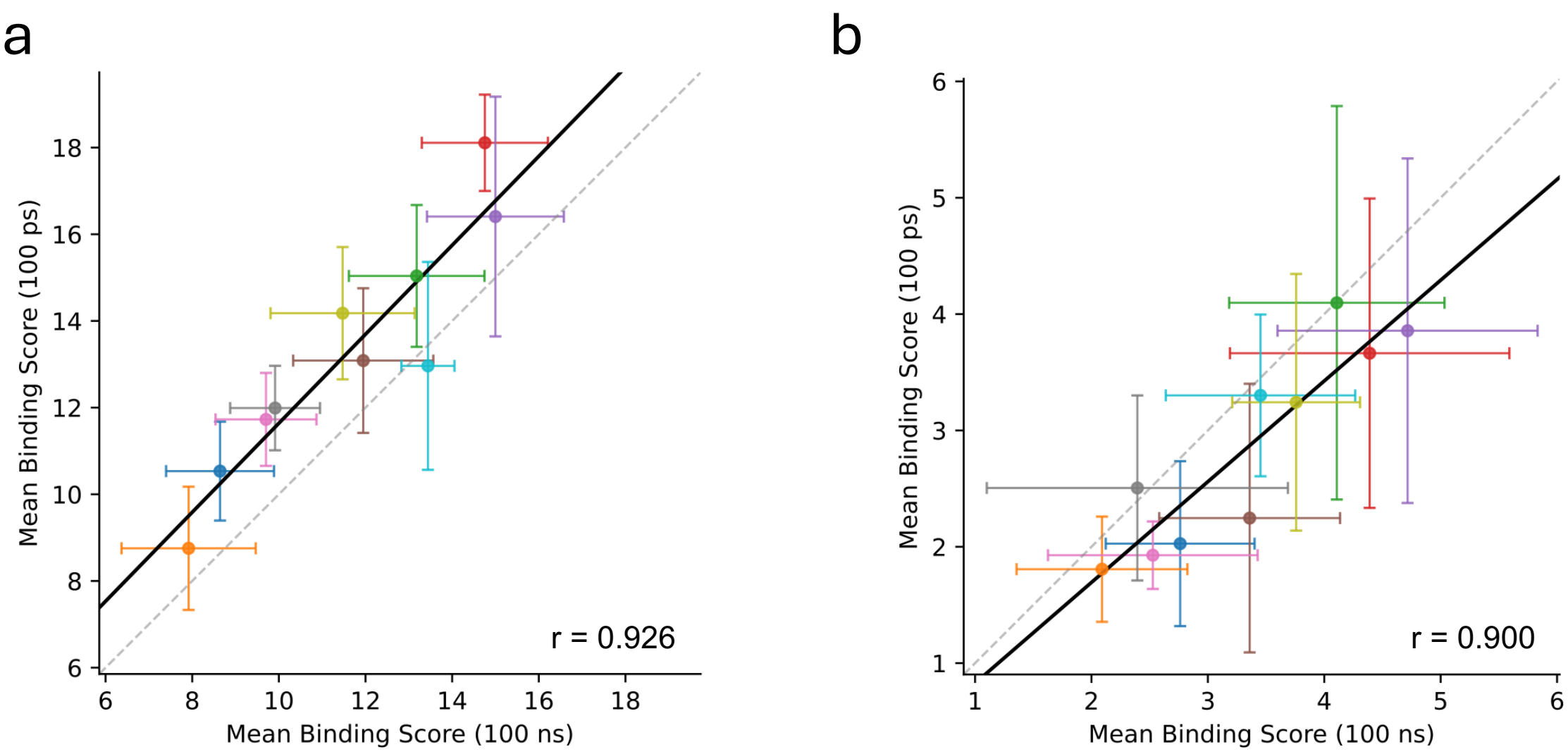

Figure S4. Comparison of binding scores between the 100 ps screening condition and the 100 ns reference condition.
Binding scores from the 100 ps screening condition and the 100 ns reference condition are shown for the 10 peptides selected from the candidates evaluated during the optimization loop (S-01 to S-10). Each point represents the mean binding score of one peptide across replicate simulations initiated from different initial structures. Error bars indicate ± 1 standard deviation across replicate simulations for the same peptide–receptor pair. (a) CXCR4. The 100 ns simulations were performed in a membrane-embedded system, whereas the 100 ps simulations were performed in water. Pearson's correlation was $r = 0.926$ ($p = 1.22 \times 10^{-4}$), and Spearman's correlation was $\rho = 0.903$ ($p = 3.44 \times 10^{-4}$). (b) NRP1. Both the 100 ps and 100 ns simulations were performed in water. Pearson's correlation was $r = 0.900$ ($p = 3.88 \times 10^{-4}$), and Spearman's correlation was $\rho = 0.879$ ($p = 8.14 \times 10^{-4}$).

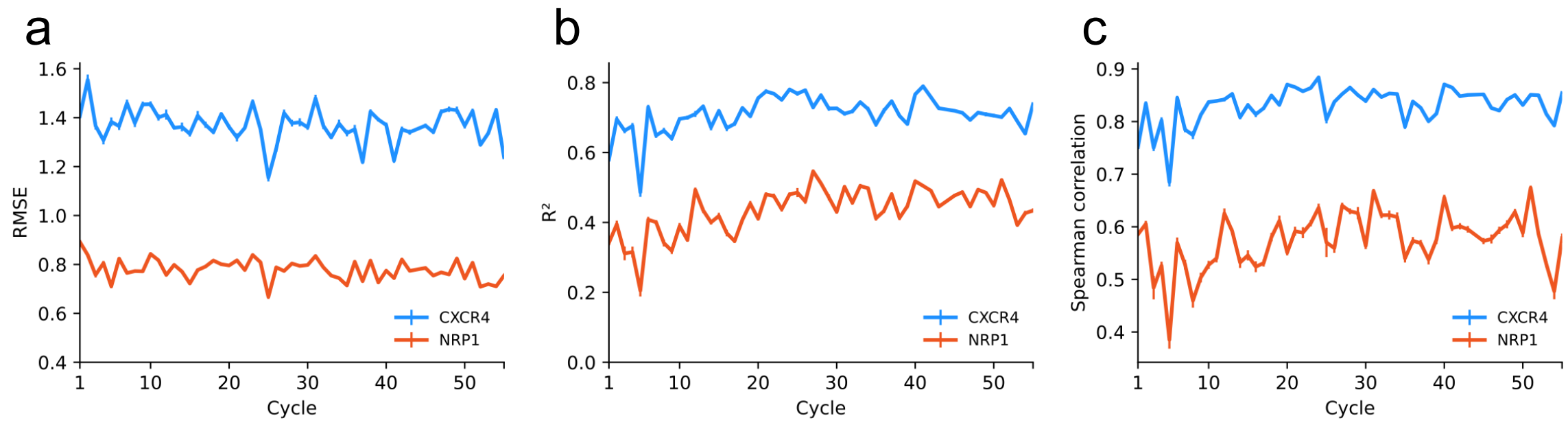

Figure S5. Validation of the surrogate model across consecutive optimization cycles.
Surrogate model performance was evaluated by comparing predictions from the N-th optimization cycle with the evaluated binding scores obtained in the subsequent $(N+1)$-th cycle. Lines and error bars represent the mean $\pm$ SD across 12 surrogate models. (a) Root mean squared error (RMSE). (b) Coefficient of determination (R^2). (c) Spearman correlation coefficient.

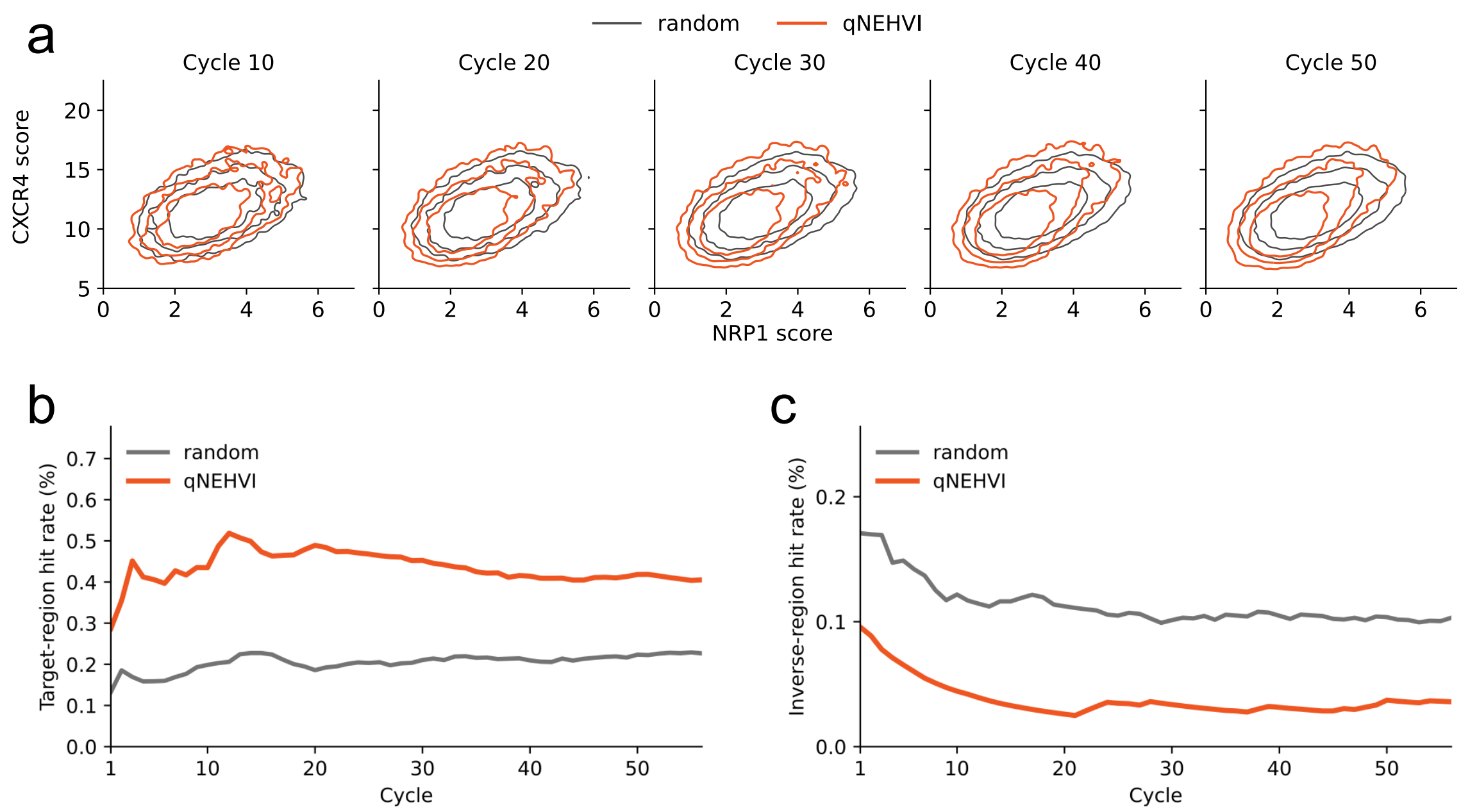

Figure S6. Comparison of qNEHVI-based and random exploration.
Comparison of peptide exploration performance between qNEHVI-based optimization and random selection. (a) Contour plots showing the cumulative distributions of evaluated CXCR4 and NRP1 binding scores up to cycles 10, 20, 30, 40, and 50. Gray: random selection; orange: qNEHVI-based selection. (b) Cumulative percentage of evaluated peptides up to each cycle that fall within the target region. (c) Cumulative percentage of evaluated peptides up to each cycle that fall within the inverse target region.

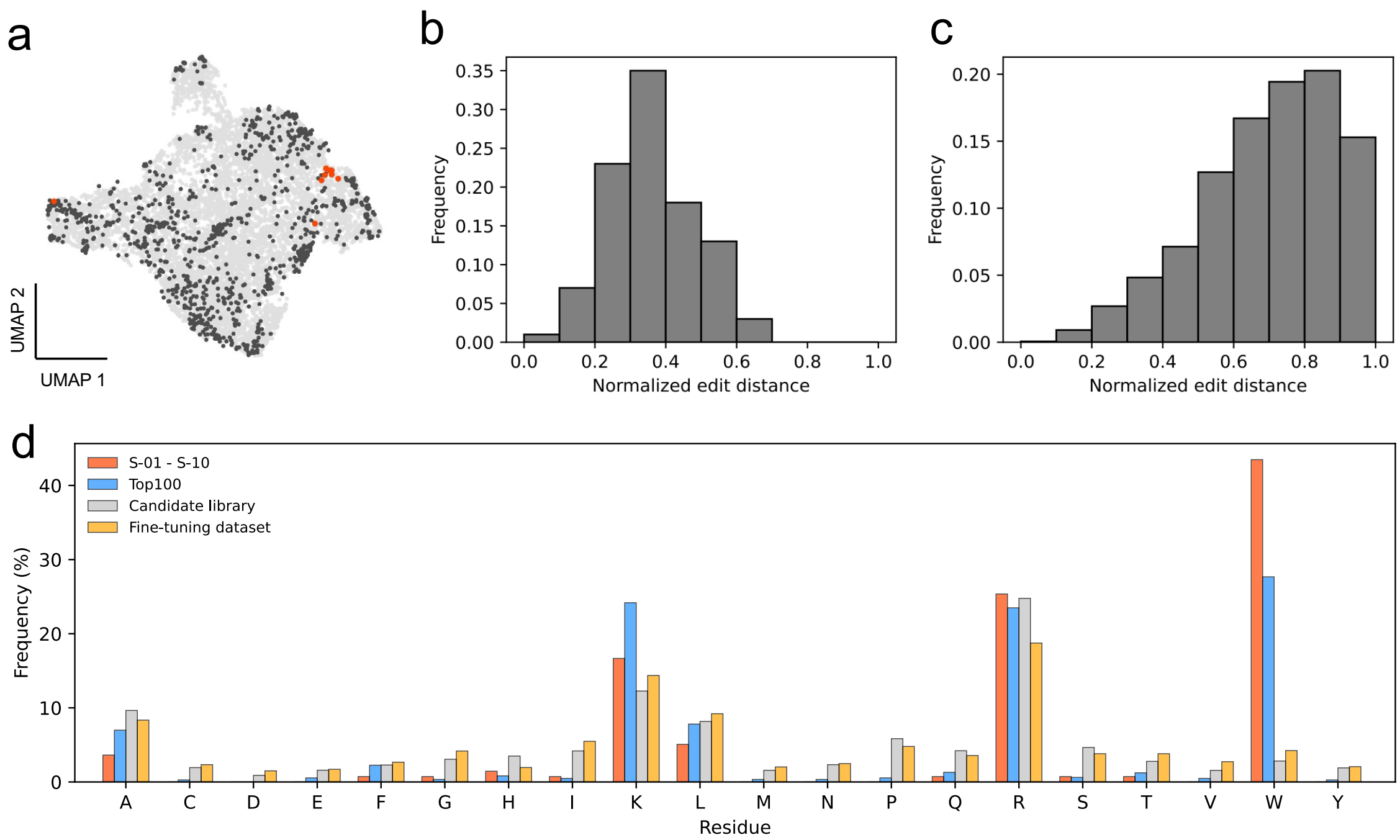

Figure S7. Sequence analysis of optimized candidates.
(a) UMAP projection of the CPP-like candidate library, the peptide sequences used for model fine-tuning, and the optimized peptides S-01–S-10. Red: S-01–S-10; dark gray: peptide sequences used for model fine-tuning; light gray: CPP-like candidate library. (b) Distribution of normalized edit distances between the top 100 sequences and the peptide sequences used for model fine-tuning. (c) Distribution of pairwise normalized edit distances among the top 100 sequences obtained through optimization. (d) Amino acid residue frequency distributions for S-01–S-10, the top 100 sequences, the candidate library, and the peptide sequences used for model fine-tuning.

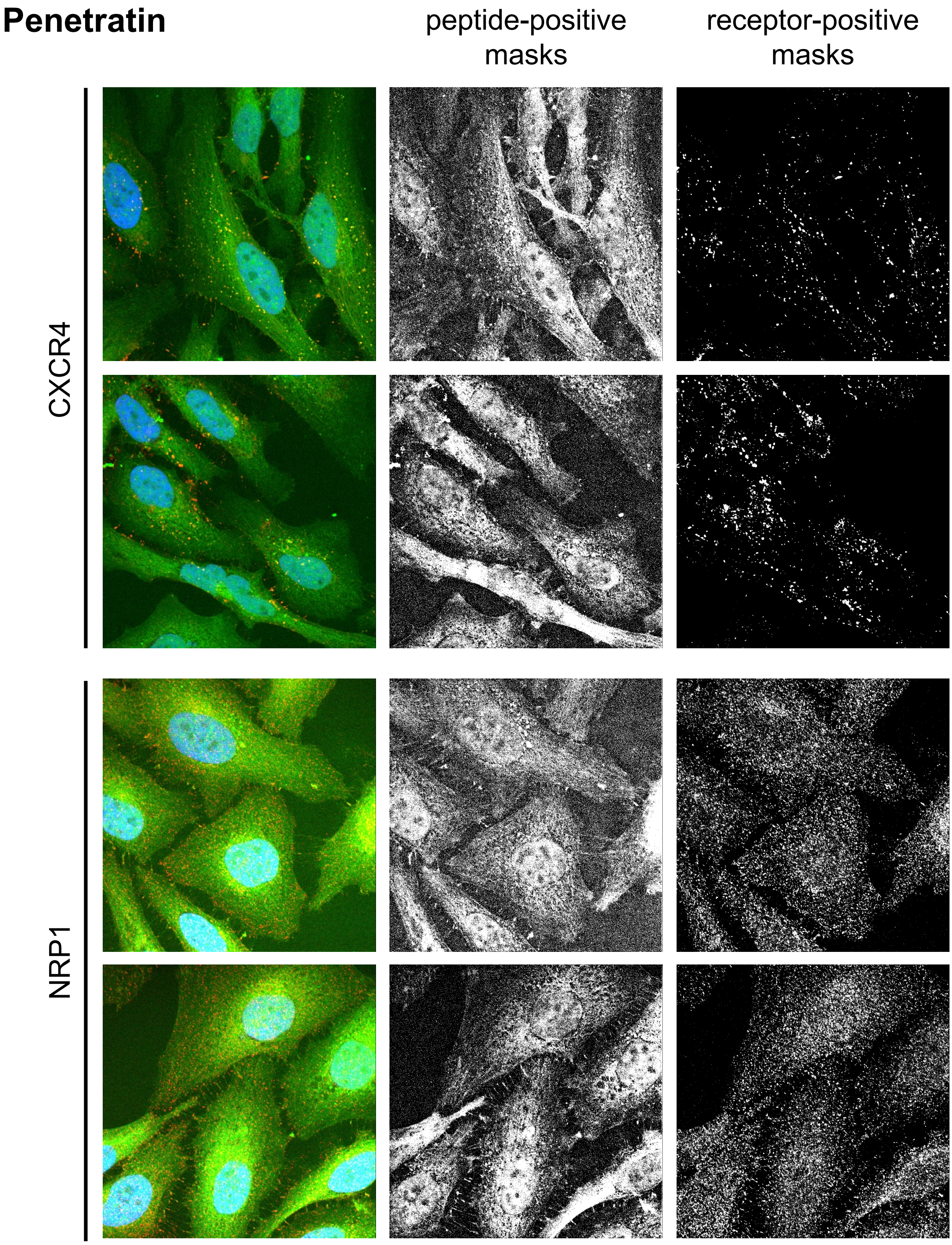

Figure S8. Representative confocal images and segmentation masks for Penetratin.
HeLa cells were treated with 5-FAM-labeled Penetratin and immunostained for CXCR4 or NRP1. Top two rows: CXCR4 condition. Bottom two rows: NRP1 condition. Left: raw confocal images (Green: Penetratin; Red: receptor; Blue: DAPI). Center: peptide-positive masks. Right: receptor-positive masks.

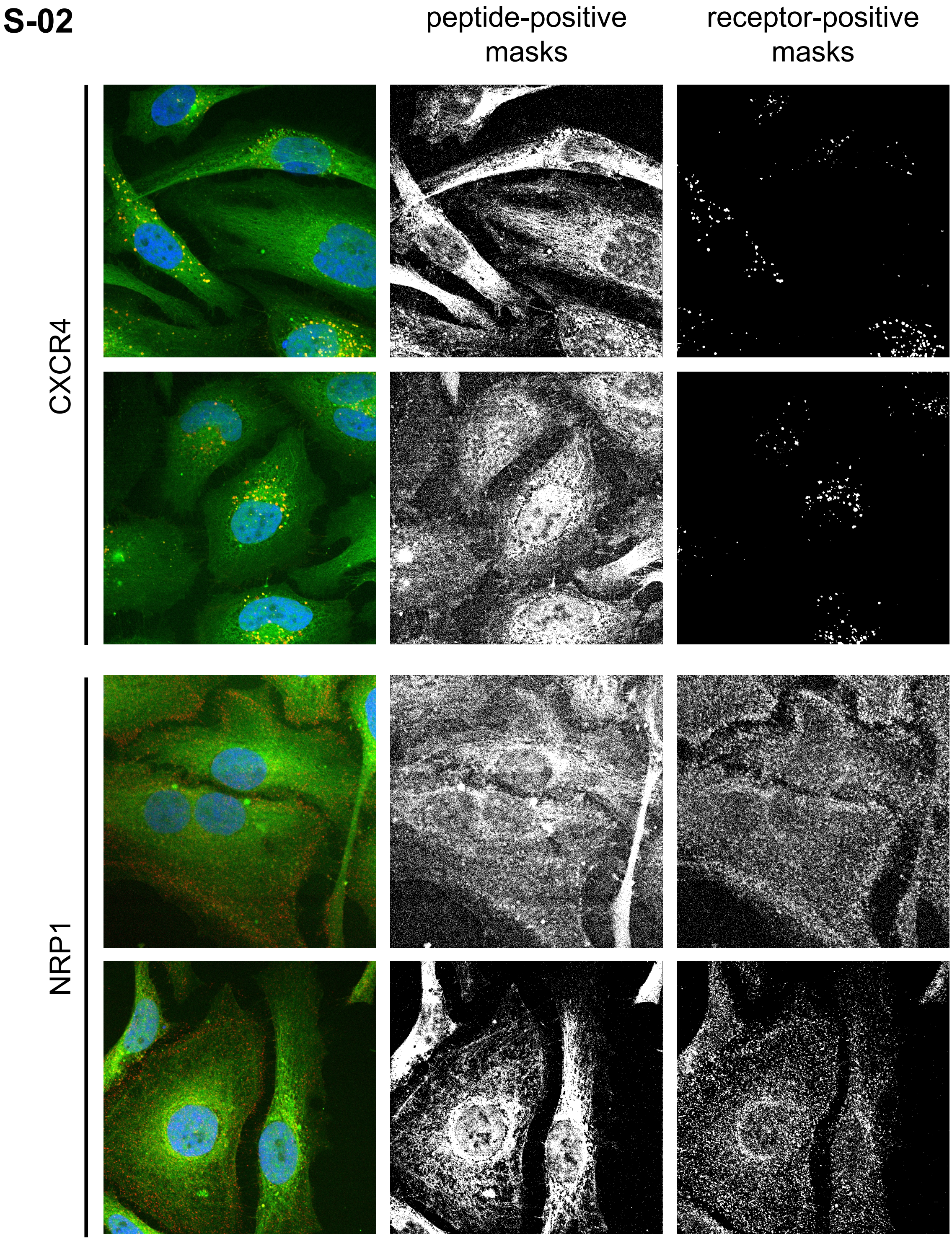

Figure S9. Representative confocal images and segmentation masks for peptide S-02.
HeLa cells were treated with 5-FAM-labeled S-02 and immunostained for CXCR4 or NRP1. Top two rows: CXCR4 condition. Bottom two rows: NRP1 condition. Left: raw confocal images (Green: S-02; Red: receptor; Blue: DAPI). Center: peptide-positive masks. Right: receptor-positive masks.

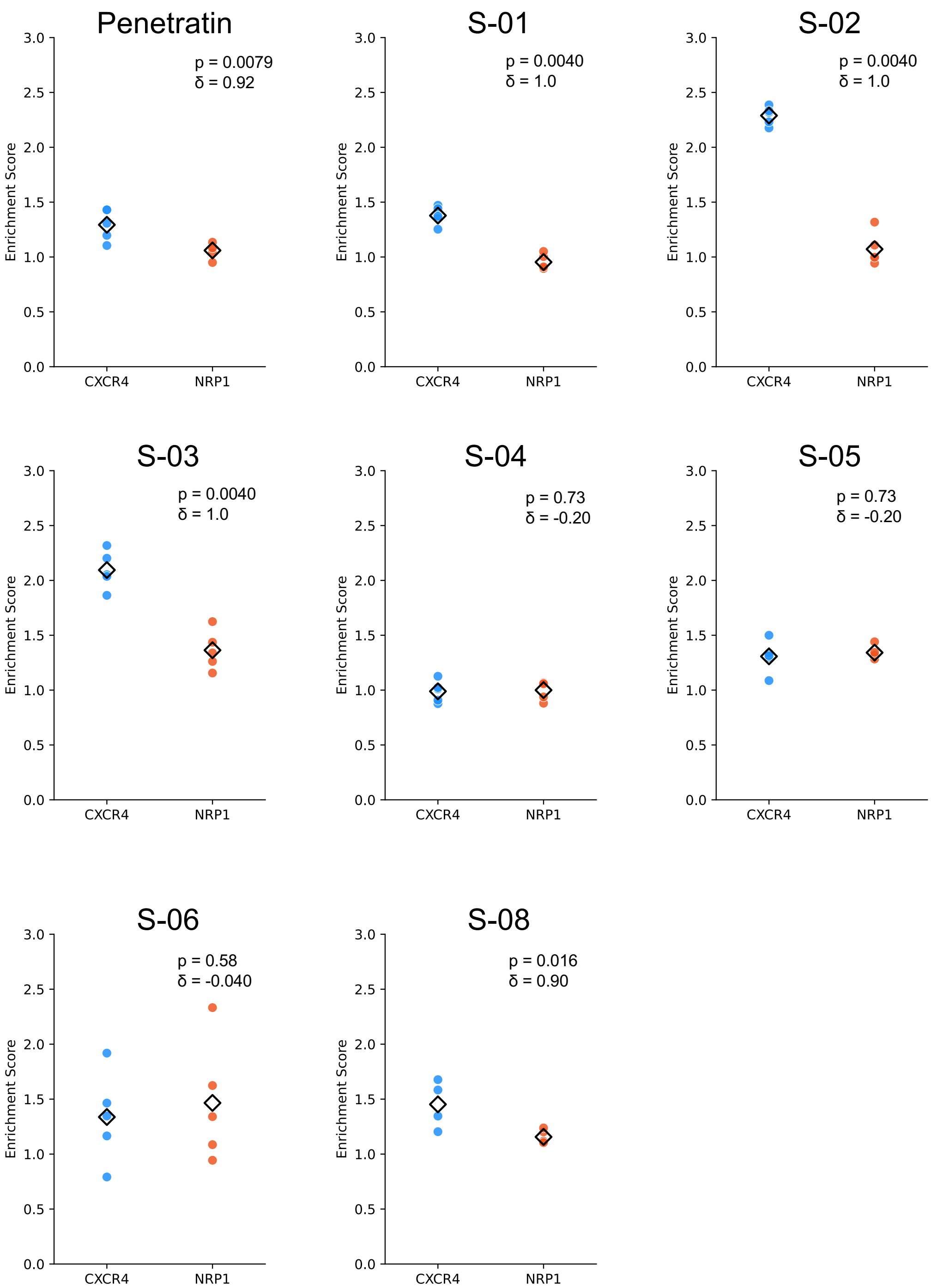

Figure S10. Receptor-associated enrichment scores for selected peptides.
Quantitative comparison of enrichment scores for Penetratin and selected peptides (S-01 to S-06, S-08). TAT, R8, S-07, S-09, and S-10 were excluded because fluorescence intensity was insufficient for reliable quantification. Each dot represents a single field of view, and diamonds indicate the mean. Panel annotations show one-sided Mann-Whitney U test p values for the comparison CXCR4 $>$ NRP1 and Cliff's δ.

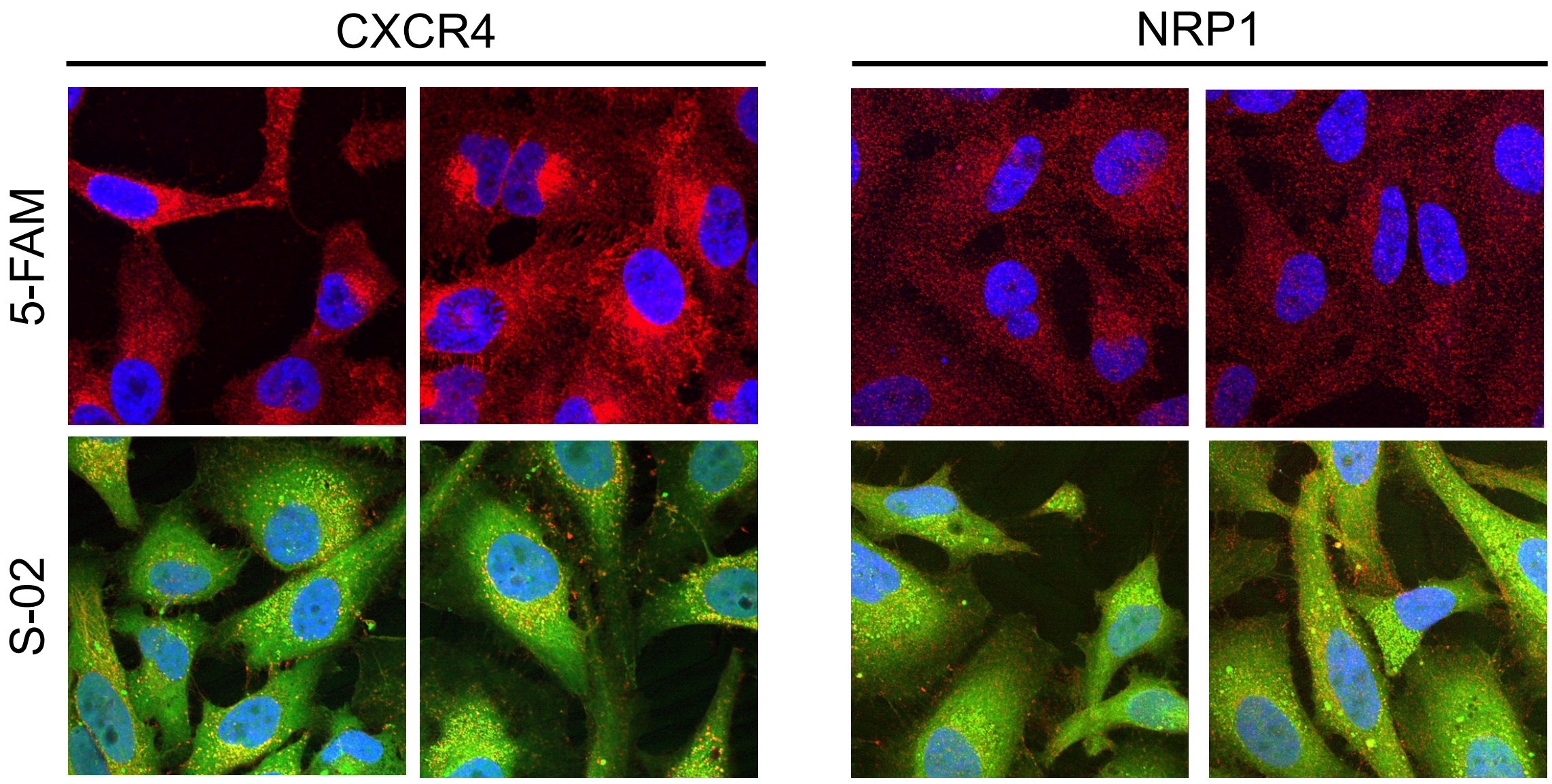

Figure S11. Free 5-FAM control images.
Representative confocal images for the free 5-FAM and 5-FAM-labeled S-02 conditions. Upper row: free 5-FAM. Lower row: 5-FAM-labeled S-02. Left: CXCR4 immunostaining condition. Right: NRP1 immunostaining condition. Green: 5-FAM signal; red: receptor signal; blue: DAPI.

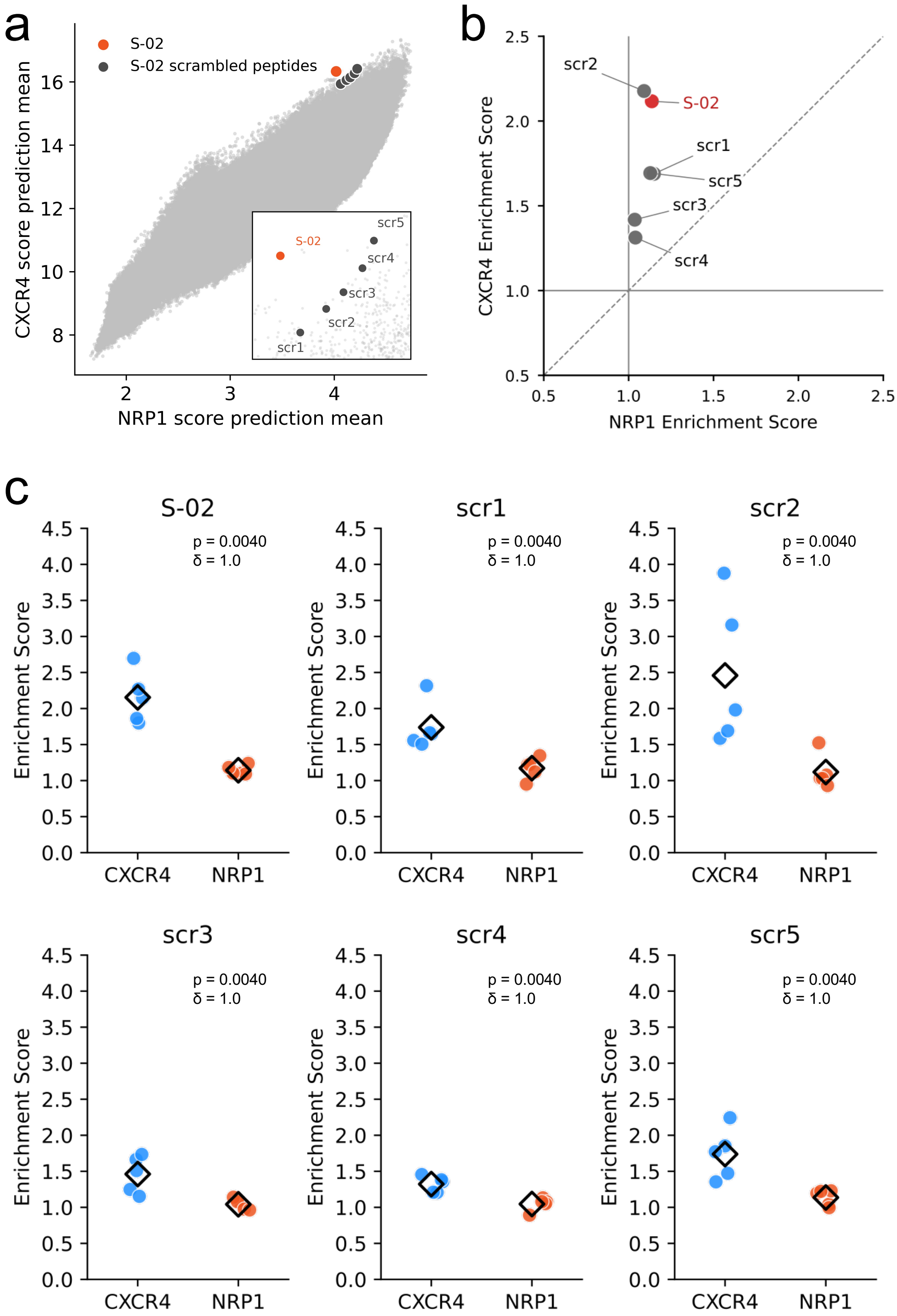

Figure S12. Scrambled peptide analysis of S-02.
Comparison of S-02 and five scrambled variants with the same amino-acid composition. (a) CXCR4 and NRP1 scores predicted by the surrogate model for the candidate library, with S-02 and the five scrambled peptides highlighted. The inset shows an enlarged view of these peptides. (b) Mean enrichment scores for S-02 and the scrambled peptides with respect to CXCR4-positive and NRP1-positive regions. (c) Enrichment score distributions for S-02 and the scrambled peptides. P values were computed using a one-sided Mann-Whitney U test, and the corresponding effect sizes are indicated as Cliff's δ.

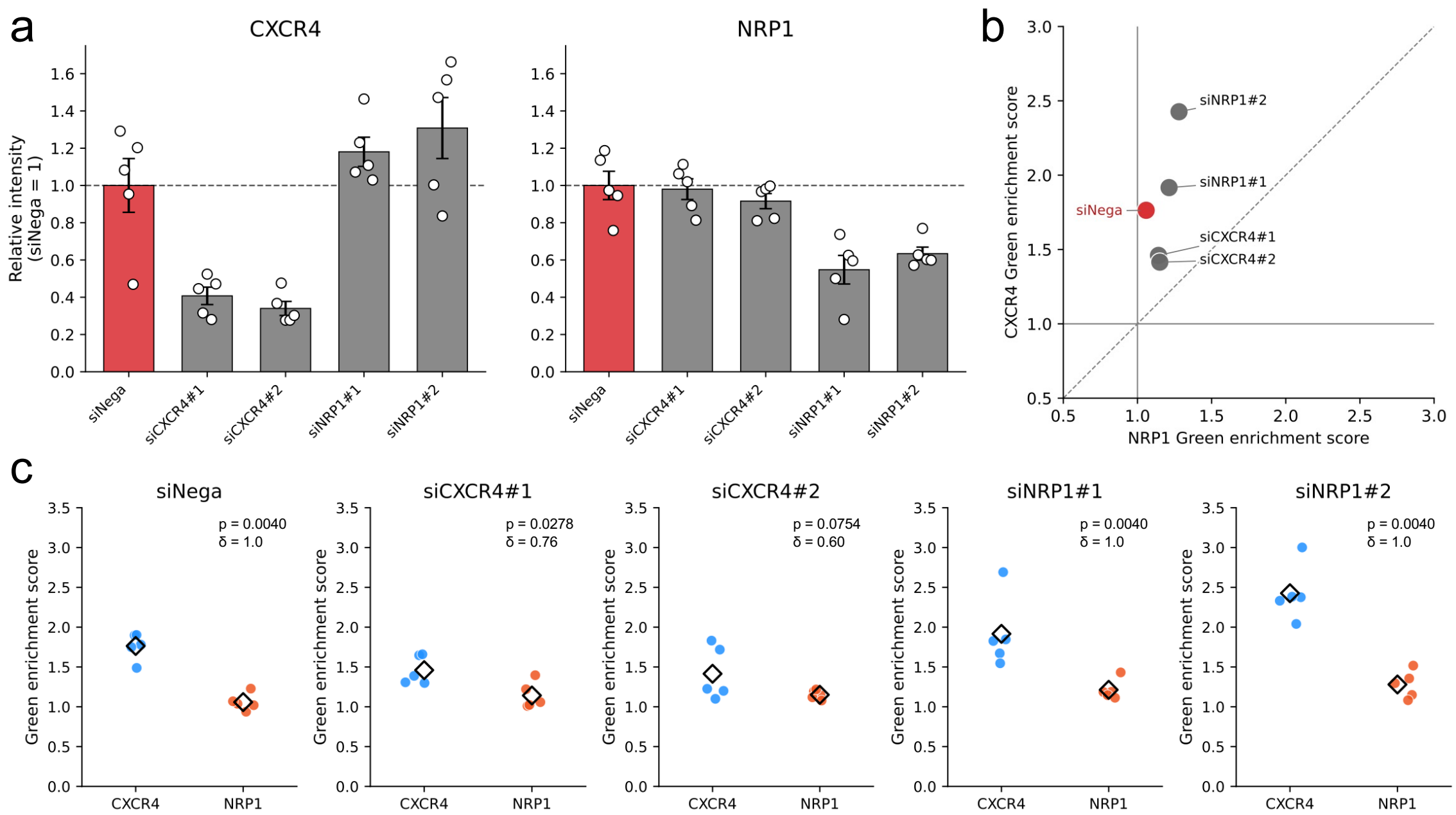

Figure S13. siRNA-mediated receptor knockdown analysis.

Analysis under negative-control siRNA (siNega), CXCR4 siRNA, and NRP1 siRNA conditions. (a) Relative receptor fluorescence intensity quantified from immunostaining images and normalized to the negative-control siRNA condition (siNega = 1). Left: CXCR4; right: NRP1. (b) Mean enrichment scores for S-02 with respect to CXCR4-positive and NRP1-positive regions under each siRNA condition. (c) Enrichment score distributions for S-02 under each siRNA condition. P values were computed using a one-sided Mann-Whitney U test, and the corresponding effect sizes are indicated as Cliff's δ.

Table S1. Architecture and training hyperparameters of the deep ensemble models.

Model	Hidden dim	Kernel	Dilation	Repeat	Learning rate	Dropout probability	Patience
1	64	$3/5$	$1/2$	2	1×10^{-5}	0.10	200
2	64	$3/5$	$2/3$	3	5×10^{-6}	0.05	100
3	128	$5/7$	$1/1$	3	1×10^{-6}	0.05	100

Table S2. Image-processing parameters used for enrichment-score quantification in Fiji.

Category	Parameter	Value
Channel assignment	Peptide channel	Channel 2
	Receptor channel	Channel 3
Image tiling	Field of view division	2×2 quadrants
Background subtraction	Rolling-ball radius	50 pixels (approximately 5.18 µm)
Peptide-positive ROI (peptide channel)	Thresholding method	Huang dark
	Minimum particle size	20 pixels^2
Receptor ROI (receptor channel)	Thresholding method	Otsu dark
	Minimum particle size	0.1 pixels^2
	Circularity	0.00-1.00
Quantification	I_{Peptide}	Mean peptide intensity in peptide-positive ROI
	I_{Receptor}	Mean peptide intensity in receptor-positive ROI
	Enrichment score	$ES = I_{\mathrm{Receptor}}/I_{\mathrm{Peptide}}$

Supplementary References

1. Hyejin Park, Jung-Hyun Park, Min Seok Kim, Kwangmin Cho, and Jae-Min Shin. In silico screening and optimization of cell-penetrating peptides using deep learning methods. *Biomolecules*, 13(3):522, 03 2023. ISSN 2218-273X. doi: 10.3390/biom13030522. URL https://www.mdpi.com/2218-273X/13/3/522.
2. Melanie P. Wescott, Irina Kufareva, Cheryl Paes, Jason R. Goodman, Yana Thaker, Bridget A. Puffer, Eli Berdougo, Joseph B. Rucker, Tracy M. Handel, and Benjamin J. Doranz. Signal transmission through the CXC chemokine receptor 4 (CXCR4) transmembrane helices. *Proceedings of the National Academy of Sciences*, 113(35):9928–9933, 08 2016. doi: 10.1073/pnas.1601278113. URL https://www.pnas.org/doi/10.1073/pnas.1601278113.
3. James L. Daly, Boris Simonetti, Katja Klein, Kai-En Chen, Maia Kavanagh Williamson, Carlos Antón-Plágaro, Deborah K. Shoemark, Lorena Simón-Gracia, Michael Bauer, Reka Hollandi, Urs F. Greber, Peter Horvath, Richard B. Sessions, Ari Helenius, Julian A. Hiscox, Tambet Teesalu, David A. Matthews, Andrew D. Davidson, Brett M. Collins, Peter J. Cullen, and Yohei Yamauchi. Neuropilin-1 is a host factor for SARS-CoV-2 infection. *Science*, 370(6518):861–865, 11 2020. doi: 10.1126/science.abd3072. URL https://www.science.org/doi/10.1126/science.abd3072.
4. Mikhail A. Lomize, Irina D. Pogozheva, Hyeon Joo, Henry I. Mosberg, and Andrei L. Lomize. OPM database and PPM web server: Resources for positioning of proteins in membranes. *Nucleic Acids Research*, 40(D1):D370–D376, 01 2012. ISSN 0305-1048. doi: 10.1093/nar/gkr703. URL https://doi.org/10.1093/nar/gkr703.
5. Andrei L. Lomize, Spencer C. Todd, and Irina D. Pogozheva. Spatial arrangement of proteins in planar and curved membranes by PPM 3.0. *Protein Science : A Publication of the Protein Society*, 31(1):209–220, 01 2022. ISSN 0961-8368. doi: 10.1002/pro.4219. URL https://pmc.ncbi.nlm.nih.gov/articles/PMC8740824/.
6. Emilia L. Wu, Xi Cheng, Sunhwan Jo, Huan Rui, Kevin C. Song, Eder M. Dávila-Contreras, Yifei Qi, Jumin Lee, Viviana Monje-Galvan, Richard M. Venable, Jeffery B. Klauda, and Wonpil Im. CHARMM-GUI Membrane Builder toward realistic biological membrane simulations. *Journal of Computational Chemistry*, 35(27):1997–2004, 2014. ISSN 1096-987X. doi: 10.1002/jcc.23702. URL https://onlinelibrary.wiley.com/doi/abs/10.1002/jcc.23702.
7. Sunhwan Jo, Joseph B. Lim, Jeffery B. Klauda, and Wonpil Im. CHARMM-GUI Membrane Builder for Mixed Bilayers and Its Application to Yeast Membranes. *Biophysical Journal*, 97(1):50–58, 07 2009. ISSN 0006-3495, 1542-0086. doi: 10.1016/j.bpj.2009.04.013. URL https://www.cell.com/biophysj/abstract/S0006-3495(09)00791-7.
8. Samuel Daulton, Maximilian Balandat, and Eytan Bakshy. Parallel bayesian optimization of multiple noisy objectives with expected hypervolume improvement. In M. Ranzato, A. Beygelzimer, Y. Dauphin, P.S. Liang, and J. Wortman Vaughan, editors, *Advances in Neural Information Processing Systems*, volume 34, pages 2187–2200. Curran Associates, Inc., 2021. URL https://proceedings.neurips.cc/paper_files/paper/2021/file/11704817e347269b7254e744b5e22dac-Paper.pdf.
